# Innovative Non-PrP-Targeted Drug Strategy Designed to Enhance Prion Clearance

**DOI:** 10.1021/acs.jmedchem.2c00205

**Published:** 2022-06-30

**Authors:** Arianna Colini Baldeschi, Marco Zattoni, Silvia Vanni, Lea Nikolic, Chiara Ferracin, Giuseppina La Sala, Maria Summa, Rosalia Bertorelli, Sine Mandrup Bertozzi, Gabriele Giachin, Paolo Carloni, Maria Laura Bolognesi, Marco De Vivo, Giuseppe Legname

**Affiliations:** † Laboratory of Prion Biology, Department of Neuroscience, Scuola Internazionale Superiore di Studi Avanzati (SISSA), Via Bonomea 265, 34136 Trieste, Italy; ‡ Molecular Modeling & Drug Discovery Lab, Istituto Italiano di Tecnologia, Via Morego 30, 16163 Genoa, Italy; § Translational Pharmacology, Istituto Italiano di Tecnologia, Via Morego 30, 16163 Genoa, Italy; ∥ Analytical Chemistry Lab, Istituto Italiano di Tecnologia, Via Morego 30, 16163 Genoa, Italy; ⊥ Department of Chemical Sciences (DiSC), University of Padua, Via F. Marzolo 1, 35131 Padova, Italy; # Institute for Advanced Simulations (IAS)-5/Institute for Neuroscience and Medicine (INM)-9, “Computational Medicine”, Forschungszentrum Jülich, 52428 Jülich, Germany; ¶ Institute for Neuroscience and Medicine (INM)-11, “Molecular Neuroscience and Neuroimaging”, Forschungszentrum Jülich, 52428 Jülich, Germany; ∇ Department of Physics, RWTH-Aachen University, 52074 Aachen, Germany; ○ Department of Pharmacy and Biotechnology, University of Bologna, Via Belmeloro 6, 40126 Bologna, Italy

## Abstract

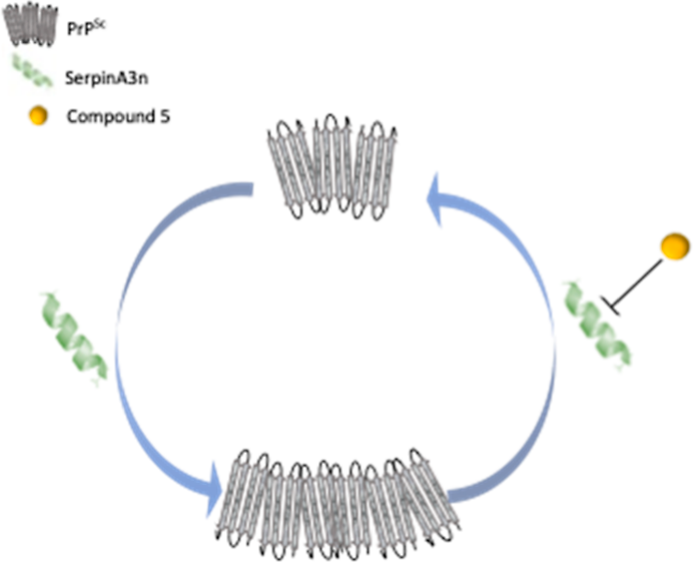

Prion diseases are a group of neurodegenerative disorders characterized by the accumulation of misfolded prion protein (called PrP^Sc^). Although conversion of the cellular prion protein (PrP^C^) to PrP^Sc^ is still not completely understood, most of the therapies developed until now are based on blocking this process. Here, we propose a new drug strategy aimed at clearing prions without any direct interaction with neither PrP^C^ nor PrP^Sc^. Starting from the recent discovery of SERPINA3/SerpinA3n upregulation during prion diseases, we have identified a small molecule, named compound 5 (ARN1468), inhibiting the function of these serpins and effectively reducing prion load in chronically infected cells. Although the low bioavailability of this compound does not allow *in vivo* studies in prion-infected mice, our strategy emerges as a novel and effective approach to the treatment of prion disease.

## Introduction

Prion diseases, or transmissible spongiform encephalopathies (TSEs), are a class of rare and fatal infectious neurodegenerative disorders characterized by brain vacuolation, neuronal loss, and cognitive and motor impairments.^1^ Etiological agents responsible for TSEs are prions, novel infectious agents derived from the physiological membrane-bound cellular prion protein (PrP^C^).^2,3^ When PrP^C^ acquires its misshapen state (referred to as PrP^Sc^), it tends to form aggregates, imposing its anomalous structure on the benign PrP^C^ molecules. Prions thus act as corruptive seeds that initiate a chain reaction of PrP misfolding and aggregation.^4^ As prions grow, fragment, and spread, they cause neuronal loss perturbing the function of the nervous system and ultimately leading to the death of affected individuals.^5^ Despite great efforts that have been dedicated to the understanding of the precise molecular mechanisms leading to prion diseases, no effective therapies have yet been developed.^6^

Small molecule-based approaches targeting PrP^C^^7,8^ or genetic strategies aimed at reducing PrP^C^ amounts^9,10^ have been proposed to impede disease progression with limited success. Thus, several attempts have been made to identify different targets involved in prion infection. In this context, the SERPINA3 protein could play a role in prion disease pathogenesis. Indeed, in 2014, a large-scale transcriptome gene expression analysis of bovine spongiform encephalopathies (BSE)-infected cynomolgus macaques (*Macaca fascicularis*) revealed a striking upregulation of SERPINA3.^11^ This upregulation was later confirmed in human frontal cortex specimens from patients affected with different types of prion diseases, both at the protein and transcript levels.^12^ A SERPINA3/SerpinA3n (its functional murine orthologue) role in prion propagation was further corroborated by our recent observation of SerpinA3n-dependent prion accumulation changes in scrapie-infected cells.^13^ Therefore, these findings make SERPINA3 an attractive target for non-PrP-targeted anti-prion drug strategies.^14^

We report here the combined use of virtual and phenotypic screening to identify anti-prion small molecules targeting SERPINA3. To the best of our knowledge, this is the first report on a novel SERPINA3 ligand active in cellular models of the prion infection.

## Results

### Structure-Based Virtual Screening

First, we conducted a structure-based virtual screening (SBVS) campaign to identify novel SERPINA3 inhibitors. We docked an internal library of ∼15,000 drug-like and non-redundant compounds^15^ against the SERPINA3 sB/sC pocket. This pocket is located at the interface between B and C beta-sheets and found to be essential for SERPIN’s activity, in particular plasminogen activator inhibitor 1 (SERPINE1).^16^

**Figure 1:**
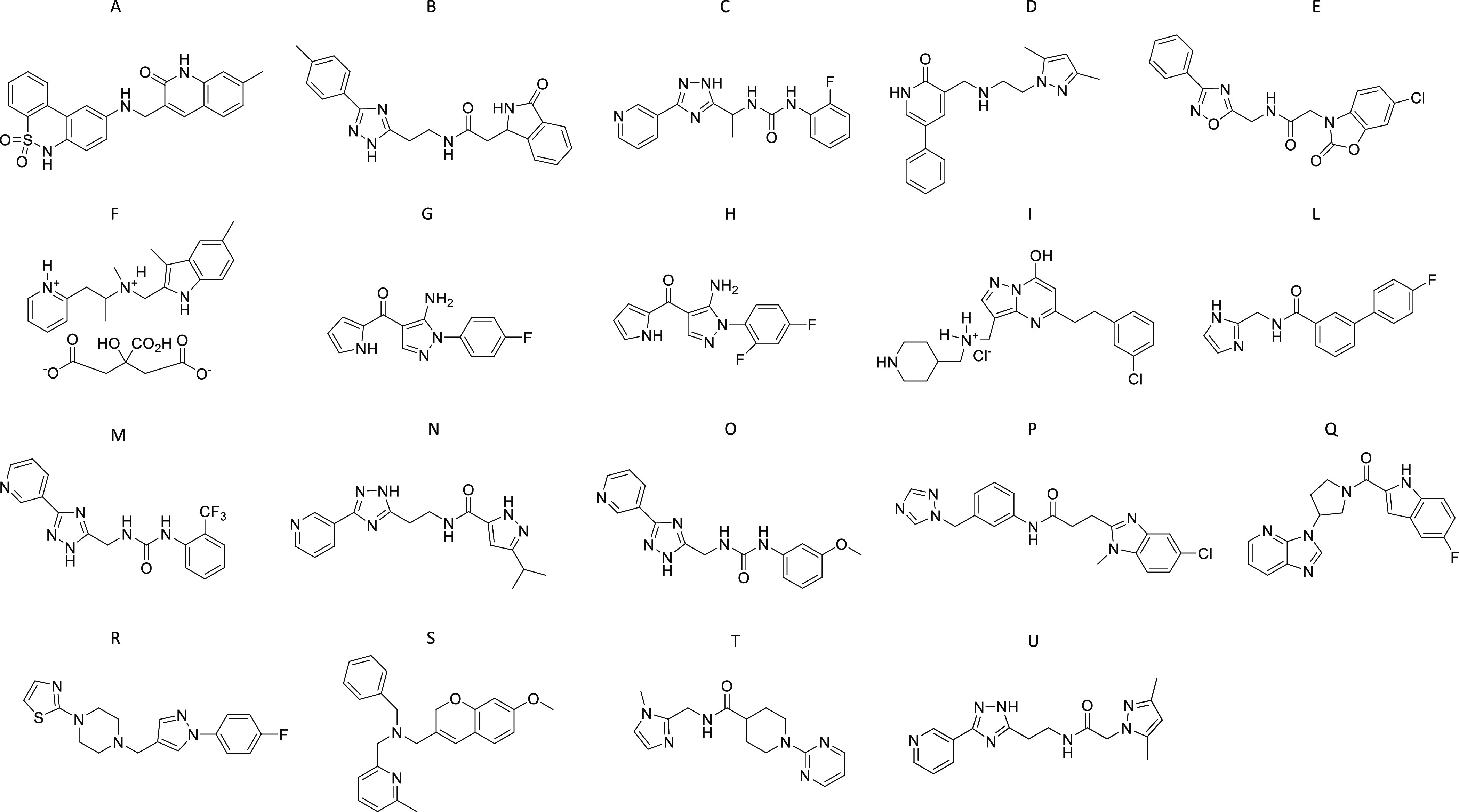
Chemical structures of compounds A–U.

Nineteen best-scoring ligands (compounds A-U) (Figure 1 and Table S1) were selected for biological assays.

### SerpinA3n Upregulation in Prion-Infected Cells

Before testing the anti-prion activity of the SERPINA3 inhibitors, the expression levels of SerpinA3n were assessed in GT1^17^ and N2a^18^ prion-infected cells. *In vitro* models of prion infection represent the most standardized and reproducible tool for studying prion propagation in an intact cellular system, since they recapitulate the key molecular events that characterize prion disease pathogenesis.^19^ Furthermore, the ease of manipulation compared to *in vivo* models makes scrapie-infected immortalized neuronal cell lines the most used model to test the efficacy of novel anti-prion therapies.^20−22^ SerpinA3n transcript was found highly upregulated in both RML- and 22L-infected GT1 (Figure S1A–C) and N2a (Figure S2A–C) cells compared to the uninfected control cells. Since SerpinA3n is a secreted protein,^23,24^ we performed a Western blot analysis of the conditioned medium collected from both prion-infected and un-infected GT1 (Figure S1D) and N2a (Figure S2D) cell lines. A statistically significant increase of the extracellular SerpinA3n, in the medium of RML- and 22L-infected GT1 (Figure S1F) and N2a cells (Figure S2F) was observed. Nevertheless, we appreciated an apparent increase in the signal intensity of the intracellular SerpinA3n bands, in both RML- and 22L-infected GT1 (Figure S1E) and N2a cells (Figure S2E), in comparison with the uninfected cells.

### Identification of SERPINA3 Small Molecule Inhibitor Showing the Highest Anti-Prion Activity

First, we determined the possible toxic effects of compounds A-U at the concentration of 20 μM by performing the MTT assay on RML-infected GT1 cell lines. We set the toxicity threshold at 70% of cell viability compared to vehicle-treated cells^21^ and discarded compound A (Figure S3A). Then, the ability of compounds to reduce PrP^Sc^ levels, in prion-infected cells, was determined by densitometric analysis of Western blot images. Relative amounts of the proteinase K (PK)-resistant PrP^Sc^ were measured in comparison with the untreated ScGT1 RML cells. We observed a marked reduction of prion accumulation after the treatment with compounds F, G, and H (Figure S4A,B). Subsequently, ScGT1 RML cell viability was again tested with a higher dose (40 μM) of compounds F, G, and H. MTT assay revealed a cytotoxic effect of compound F (Figure S3B); therefore, it was discarded from further analysis. Evaluation of the anti-prion effect of compounds G and H at 40 μM in ScGT1 RML cell lines revealed a statistically significant reduction of PrP^Sc^ levels in cell-treated with compound H (Figure 3A,B).

**Figure 2:**
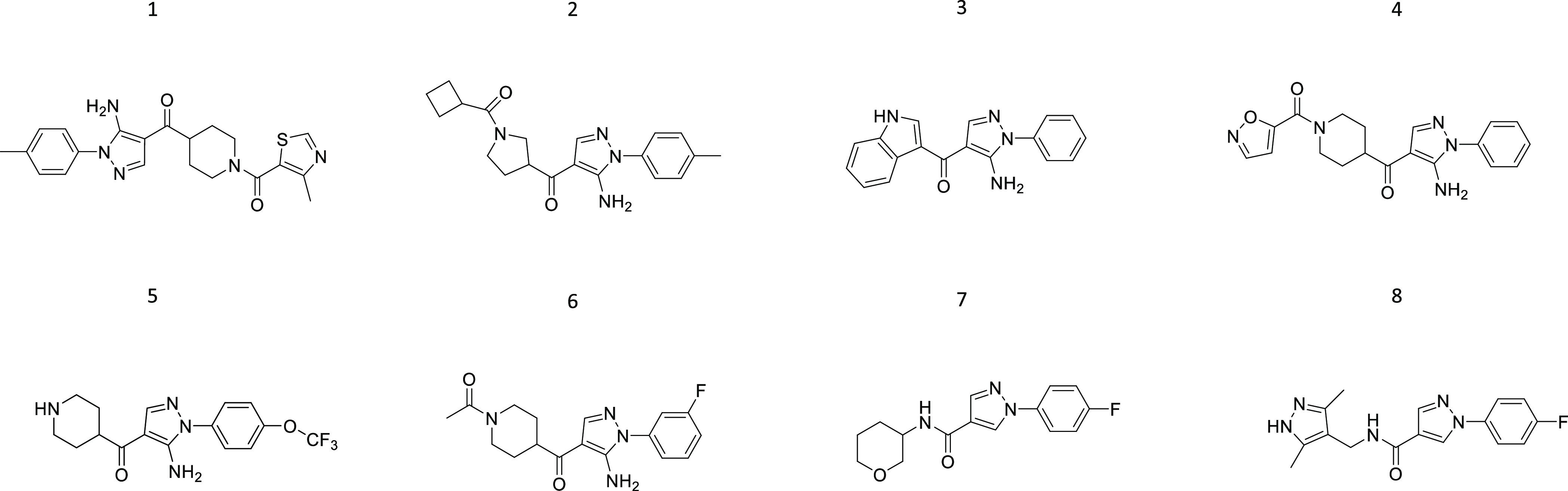
Chemical structures of compounds 1–8.

The most active compounds tested in the acute treatment experiments (i.e., G and H) share a common scaffold based on the 5-aminopyrazole core. Therefore, we decided to perform a second, more specific screening of our chemical library for further exploring new compounds bearing this heterocyclic moiety. A ligand-based similarity search of our in-house virtual library of ∼15,000 compounds resulted in the identification of eight new analogues (1–8) (Figure 2 and Table S1).^15^

**Figure 3:**
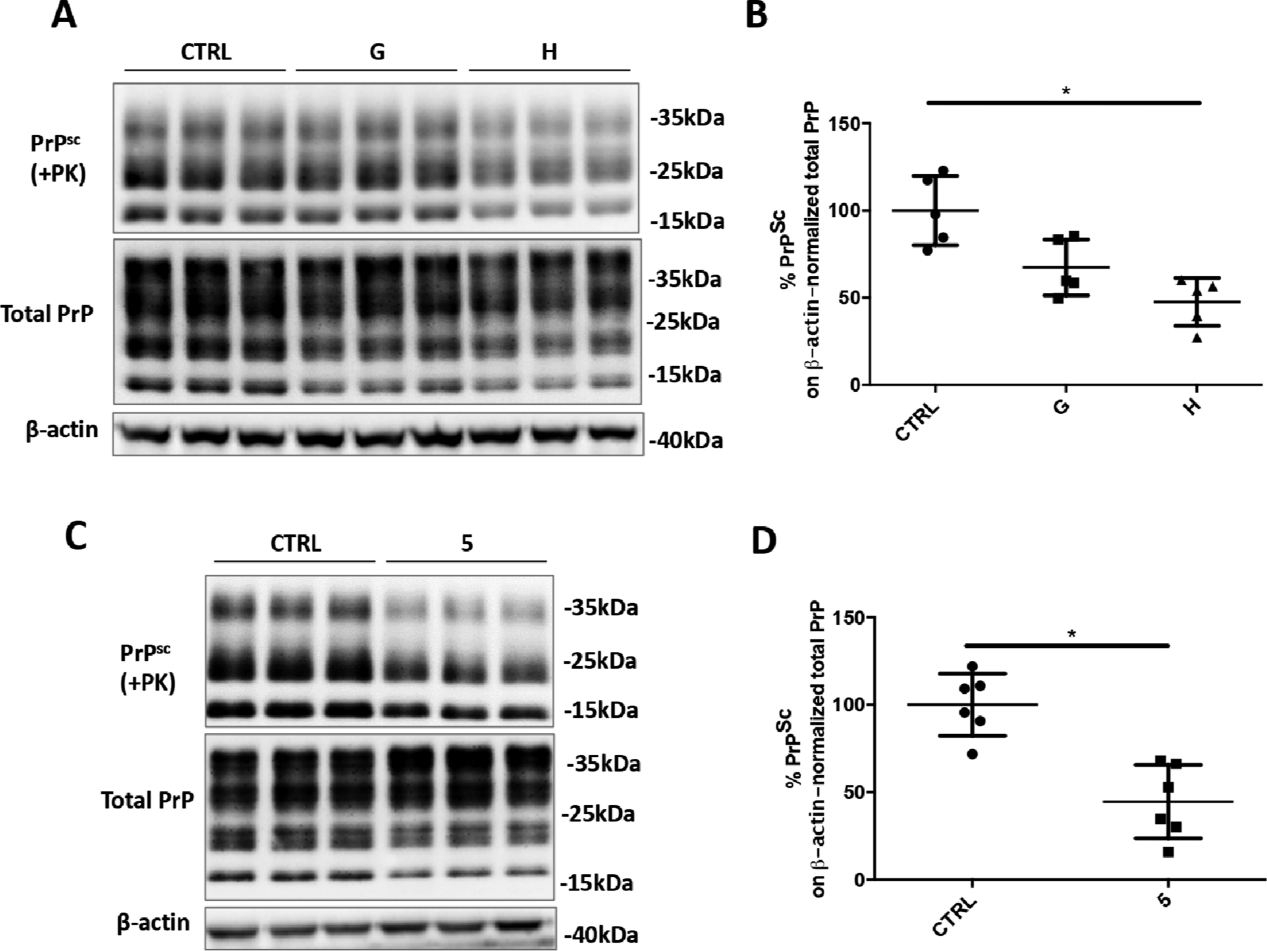
Anti-prion effect of anti-SERPINA3 hit compounds in ScGT1 RML cell lines. (A,C) Representative Western blot image of PrP^Sc^, total PrP, and β-actin in lysates from ScGT1 RML treated with vehicle (CTRL) or compounds G and H at 40 μM, or compound 5 (ARN1468) at 20 μM. Molecular weight markers are represented on the right (kDa). (B,D) Densitometric analysis of normalized PrP^Sc^ levels in ScGT1 RML treated with the vehicle or the drug. Experiments have been performed in triples (*n* = 6). Statistical significance was assessed using Friedman test and Dunn’s multiple comparison compared to control cells (B) or Wilcoxon matched-pair signed rank test (D) *p < 0.05.

MTT assay revealed compound 3 as toxic at the concentration of 20 μM in the ScGT1 RML cell line (Figure S3C). Screening of the remaining seven compounds (20 μM) highlighted a marked decrease in prion accumulation upon the treatment with compound 5 (ARN1468) (Figure S5). Its anti-prion effect was further validated in the ScGT1 RML cell line, and the observed PrP^Sc^ reduction was around 60% on average (Figure 3C,D).

### Molecular Docking and Inhibitory Activity of compound 5 Against SerpinA3n

Compound 5 was discovered as the most active compound, and its predicted binding mode is depicted in Figure S6. The 4-trifluoromethoxy-phenyl moiety fits into the small hydrophobic pocket formed by Phe198, Leu223, Leu226, Met 196, Leu242, and Trp194. While the 5-amino-pyrazole core is located at the entrance of the sB/sC pocket, the piperidine moiety is exposed to the solvent, forming an H-bond with Glu195. The ability of serpins to trap their target proteases in a covalent, SDS-resistant complex was exploited to confirm the inhibition of SerpinA3n by 5.^25^ Therefore, recombinant SerpinA3n was incubated with chymotrypsin, one of its known target proteases,^26^ together with increasing concentrations of compound 5. Coomassie blue staining of the SDS-PAGE separated samples showed a dose-dependent activity of 5, with the concentration of 1 mM completely preventing the SerpinA3n-chymotrypsin covalent complex formation (Figure S7).

Moreover, to further investigate the interaction between SerpinA3n and compound 5, the thermodynamic parameters of the binding were determined by isothermal titration calorimetry (ITC). In the ITC measurements, the protein was titrated with compound 5; both species were in 25 mM Tris, 50 mM KCl, 0.3% DMF, and pH 8.0. A representative experiment is shown in Figure S8A, where the exothermic nature of the interaction is visible. Protein concentration was limited to 16 μM since SerpinA3n showed aggregation propensity at higher concentrations. For this reason, the titration produces a saturation-shaped thermogram that does not reach a complete saturation plateau. The titration data were fitted by the sigmoidal binding isotherm of the one binding-site model (Figure S8B). From two independent experiments, we derived an apparent mean *K*_D_ of 26 ± 2.36 μM for the single macroscopic dissociation constant. The derived stoichiometry for the complex is 1:1 (1.4 ± 0.52). Preliminary analysis of the thermodynamic signature for the binding of SerpinA3n and 5 suggests a binding driven by favorable hydrogen with a contribution of hydrophobic interactions (Figure S8B, inset). For the sake of clarity, we also showed the control experiments where the buffer was titrated with 5 and SerpinA3n (Figure S8C,D, respectively) showing no binding.

### Strain and Cellular Host-Independent Anti-Prion Effect of Compound 5

**Figure 4:**
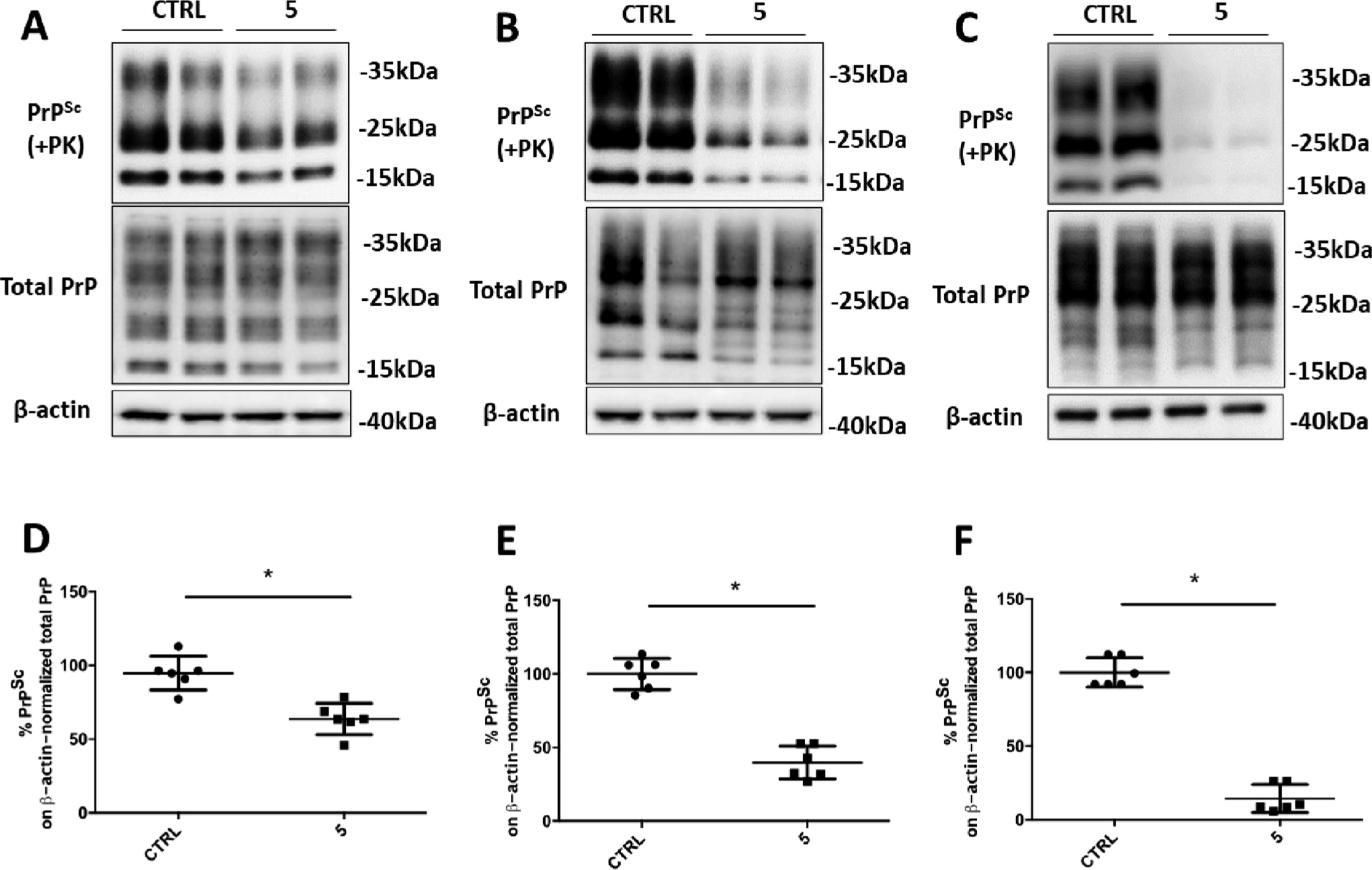
Anti-prion effect of compound 5 in ScGT1 22L, ScN2a RML, and ScN2a 22L cell lines. (A–C) Representative Western blot images of PrP^Sc^, total PrP, and β-actin in lysates from ScGT1 22L (A), ScN2a RML (B), and ScN2a 22L (C) treated with vehicle (CTRL) or compound 5 at 20 μM. Molecular weight markers are shown on the right (kDa). (D–F) Densitometric analysis of normalized PrP^Sc^ levels in ScGT1 22L (D), ScN2a RML (E), and ScN2a 22L (F) treated with the vehicle or the drug. Experiments have been performed in six times (*n* = 6). Statistical significance was assessed by Wilcoxon matched pairs signed rank test, *p < 0.05.

The anti-prion activity of 5 was also assessed in ScGT1 cells infected with the 22L strain. The treatment reduced PrP^Sc^ levels by 35%, on average (Figure 4A,D). The ScN2a RML cell line treated with compound 5 revealed an average of 60% reduction in prion load (Figure 4B,E). Additionally, 5 was tested in ScN2a cells infected with 22L prion strain where it reduced prion levels by almost 85% (Figure 4C,F).

**Figure 5:**
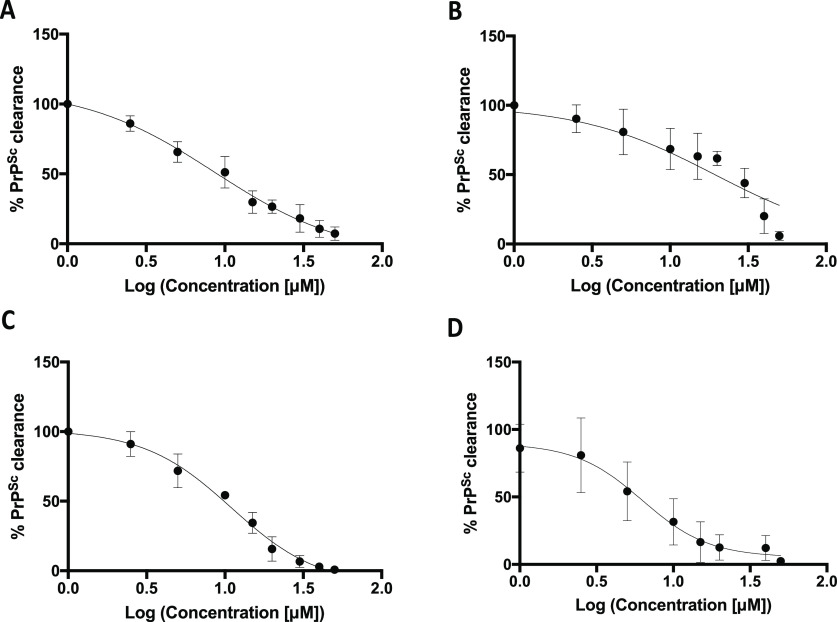
Dose–response curve of compound 5 on RML- and 22L-infected GT1 and N2a cell lines. Densitometric analysis of PrP^Sc^ level clearance after 3 days treatment with compound 5 in ScGT1 RML (A, EC_50_ = 8.64), ScGT1 22L (B, EC_50_ = 19.3), ScN2a RML (C, EC_50_ = 11.2), and ScN2a 22L (D, EC_50_ = 6.27) (for all titrations, *n* = 3).

EC_50_ of 5 in both N2a and GT1 prion-infected cell lines ranged from 6 to 19 μM (Figure 5).

Furthermore, its cytotoxicity was estimated after a 6-day treatment with LD_50_ values ranging from 34 to 55 μM (higher compared to EC_50_ values) (Figure S9).

### Chronic Treatment of Established and *de Novo* Prion-Infected Cells with Compound 5

**Figure 6:**
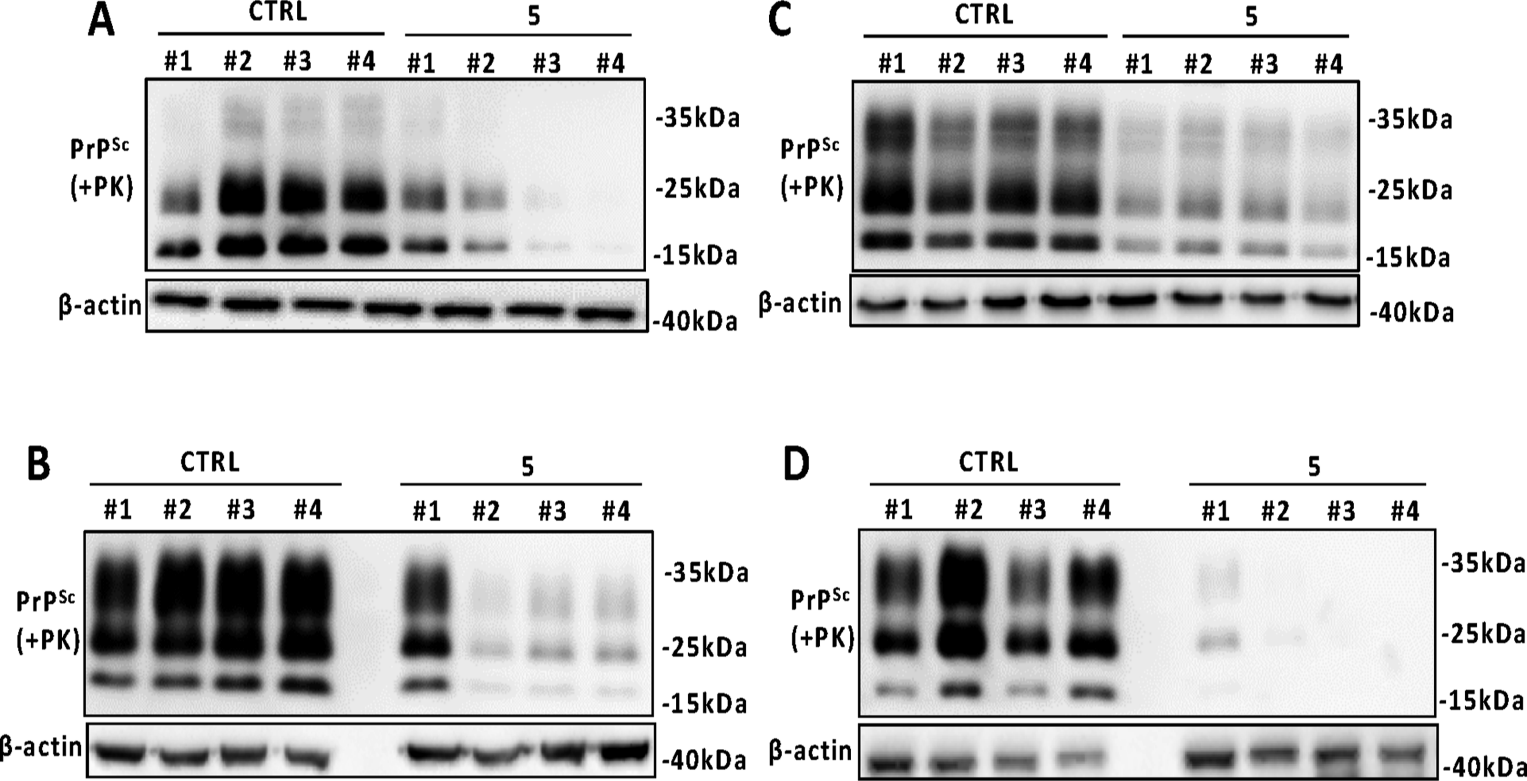
Chronic treatment of prion-infected GT1 and N2a cell line with compound 5. Western blot analysis of PrP^Sc^ in lysates from ScGT1 RML (A), ScN2a RML (B), ScGT1 22L (C), and ScN2a 22L (D) treated with vehicle or compound 5 at 20 μM for four passages (up to 1 month). β-actin was used as a loading control. Molecular weight markers are shown on the right of each Western blot (kDa).

We next chronically treated both RML- and 22L-infected ScGT1 and ScN2a cell lines with compound 5 for several passages, every 5 days of culture. PrP^Sc^ levels began to decrease since the first passage, in the presence of compound 5, and its clearance gradually increased during the subsequent passages, in ScGT1 RML (Figure 6A), ScN2a RML (Figure 6B), ScGT1 22L (Figure 6C), and ScN2a 22L (Figure 6D).

Moreover, *de novo* infection of GT1 cells with the RML strain was performed in the presence of 5 for four passages. While the appearance of prions in control *de novo*-infected GT1 cells was stable across all passages, the cells simultaneously treated with 5 showed a delay in the appearance of prion accumulation (Figure S10).

### Anti-Prion Activity of Compound 5 Does Not Affect PrP Metabolism, Localization, or Aggregation Propensity

To rule out a PrP^C^-directed anti-prion activity for compound 5, we evaluated PrP^C^ levels in compound 5-treated cells. Relative to control cells, no difference in the protein levels was observed in either GT1 (Figure S11A,B) or N2a cells (Figure S11D,E). Furthermore, no changes in cell viability were observed upon compound 5 treatment, neither in GT1 (Figure S11C) nor in N2a cells (Figure S11F). To exclude any influence of compound 5 on PrP aggregation propensity, that would be responsible for the decreased prion load in treated cells, RT-QuIC assay was performed. This technique employs cycles of shaking and incubation, recombinant PrP as the substrate for PrP^Sc^ amplification, and a thioflavin T (ThT)-based detection method.^27^ Results indicated no delay in the lag phase of the PrP aggregation process when compound 5 was added to the reaction with the PrP^Sc^ seeds and recombinant PrP. We concluded that PrP^Sc^-induced misfolding and conversion of PrP^C^ into an aggregated amyloid is not hampered by compound 5 (Figure S11G). Additionally, to test the effect of 5 on PrP^C^ localization, we performed immunofluorescence analysis on compound 5-treated N2a cells. No PrP^C^ shift toward other cellular compartments was observed in treated cells compared to the control (Figure S12).

### Compound 5 and Quinacrine Synergistic Anti-Prion Effect

**Figure 7:**
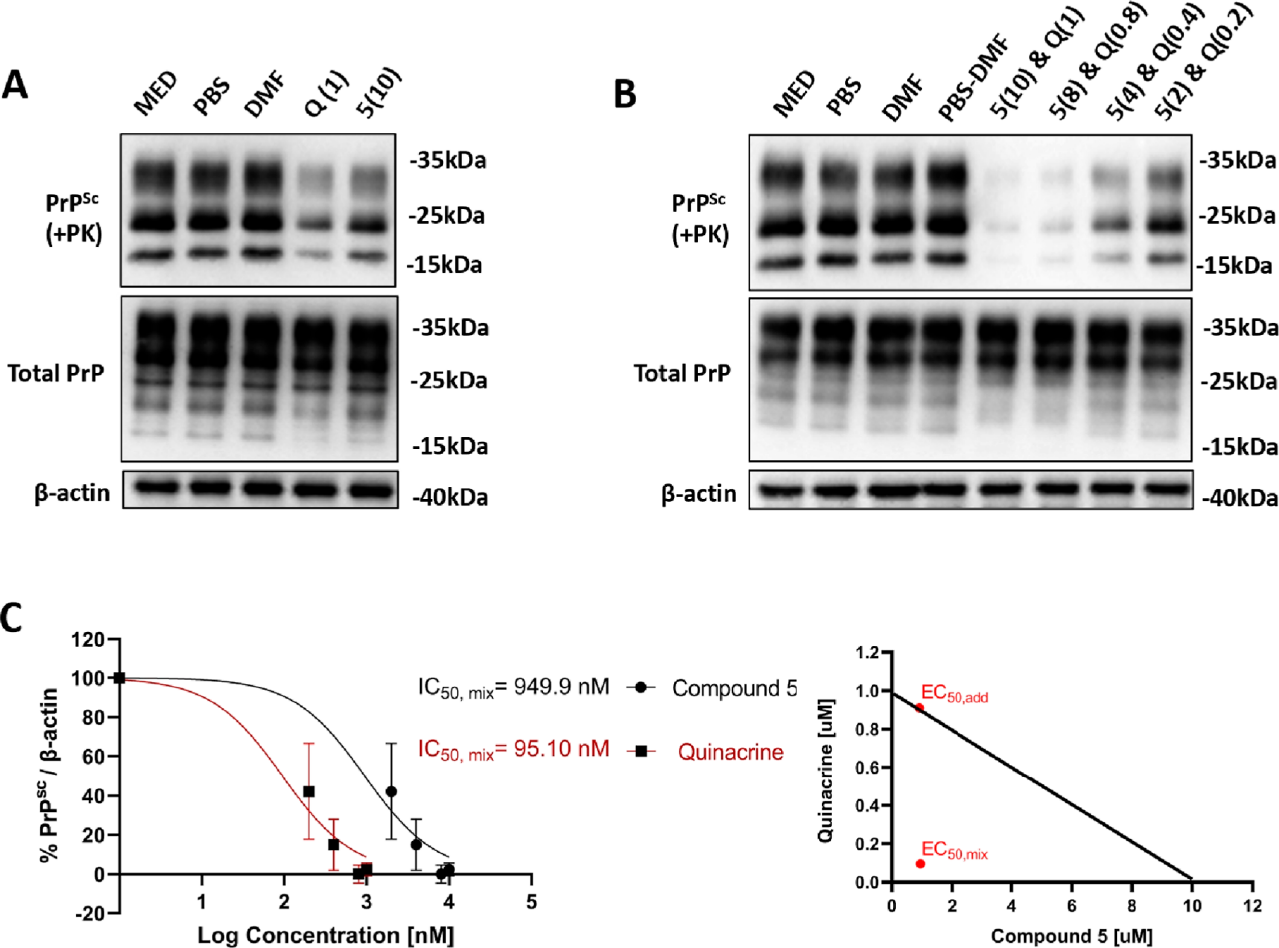
Compound 5 and quinacrine treatment of ScN2a RML cells. (A,B) Representative Western blot images of PrP^Sc^, total PrP, and β-actin in lysates from ScN2a RML cells treated with (A) vehicles, quinacrine (1 μM), or compound 5 (10 μM) and (B) vehicles or a dose gradient of compound 5 and quinacrine (2–10 μM and 0.2–1 μM, respectively). (C) Pairwise dose–response data (left) and isobologram synergy plot (right) were presented for the combination treatment. Dose–response data are derived from a nonlinear regression of 3 experimental replicates, plotted with mean ± SD. The diagonal line of the isobologram represents the additive line. EC_50,add_ dot corresponds to the calculated, theoretical paired value, when assuming an additive interaction between the two drugs. EC_50,mix_ represents the paired value of drug concentrations assessed or synergism. The interaction index (γ) was calculated, and it corresponds to a value of 0.19.

To assess the anti-prion potential of a combination treatment, we carried out a drug interaction study on ScN2a RML cells. Cells were acutely treated with a combination of compound 5 and another well-known anti-prion drug–quinacrine.^28^ Combination treatment resulted in a striking reduction of RML accumulation in the infected N2a cell line. Thus, compound 5 and quinacrine act synergically to clear prions, as revealed in the isobologram analysis (Figure 7).

### Compound 5 Pharmacokinetic Profile

**Figure 8:**
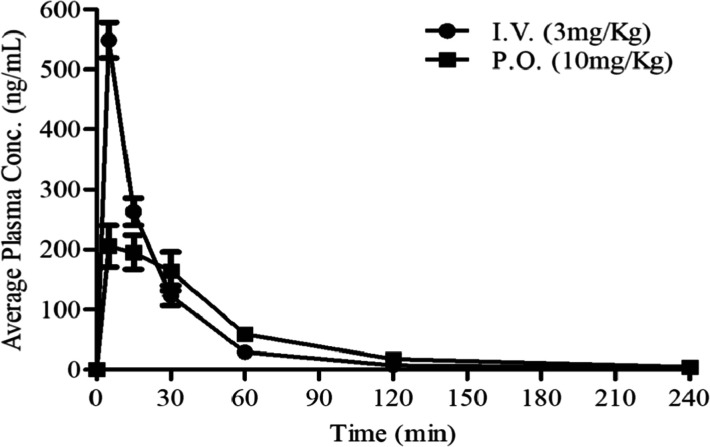
Pharmacokinetic profile of compound 5 *in vivo*. The pharmacokinetic profiles of compound 5 in plasma following intravenous (I.V.) and oral (P.O.) administration to male C57BL/6 mice (*n* = 3 per time point).

Animals that were treated by compound 5 via both intravenous (I.V.) and oral (P.O.) administration displayed normal behavior without side effects for the entire period of experiments. Administration of compound 5 showed fast and moderate absorption (*C*_max_ = 206 ng/mL P.O. and *C*_max_ = 549 ng/mL I.V. at 5 min) and high clearance in plasma (791 mL/min/kg). The exposure (AUC) over the time interval of 0–4 h was 12,192 min × ng/mL, resulting in oral bioavailability of around 26%, calculated over the same time interval (Figure 8 and Table 1). Only negligible amount (very low levels) of compound 5 was measured in the brain after I.V. and P.O. administration, very close to the limit of quantification (5 nM).

**Table 1:** Pharmacokinetic Parameters of Compound 5 Following Intravenous (I.V.) and Oral (P.O.) Administration to Male C57BL/6 Mice^a^

pharmacokinetic parameters		
parameter	I.V.	P.O.
dose (mg/Kg)	3	10
*C*_max_ [ng/mL (μM)] (obs)	549 (1.55)	206 (0.58)
*T*_max_ (min) (obs)	5	5
AUC [min × ng/mL (μM × *h*)] (calc)	14,192 (0.67)	12,192 (0.57)
*t*_1/2_ (min) (elimination phase) (calc)	98	65
*V*_D_ (mL/Kg) (calc)	29,000	74,291
CL (mL/min/Kg) (calc)	205	791

a The AUC was calculated based on the time interval *t* = 0–240 min.

## Discussion and Conclusions

Despite many efforts that were made to find a therapy for fatal neurodegenerative disorders, such as prion diseases, no cure is currently available. So far, all pharmacological and genetic approaches developed in the field directly target either PrP^C^ or PrP^Sc^.^6^ Unfortunately, none of them produced effective therapeutic results. Omics approaches are shedding light on several biological processes underlying the pathogenesis of prion diseases, therefore uncovering potential targets for the therapeutic strategies.^29^ Gene expression analysis of intracranially BSE-challenged cynomolgus macaques revealed a significant upregulation of the SERPINA3 transcript in infected animals.^11^ The mRNA up-regulation was previously observed in the central nervous system of sporadic Creutzfeldt-Jacob disease patients, while cerebrospinal fluid and urine samples revealed high levels of the SERPINA3 protein.^30^ Moreover, increased mRNA levels of its murine orthologue (SerpinA3n) have been found in different mouse models of prion disease,^31−34^ as its expression progressively increased during the course of the disease.^12,35^

Thus, we proposed a novel non-PrP targeted therapeutic strategy aimed to treat prion diseases by inhibiting SERPINA3/SerpinA3n. Since the evaluation of anti-prion compounds efficacy is prevalently performed in cell-based *in vitro* screenings,^36^ we took advantage of two immortalized cell-based models of prion infection. First, SerpinA3n expression levels were assessed in chronically infected N2a and GT1 mouse cell lines, observing a marked upregulation of both SerpinA3n transcript and protein. Then, small molecules derived from two libraries *in silico* designed to bind SERPINA3 were tested for their ability to reduce prion amounts in the RML-infected GT1 cells. Among all tested small molecules, a marked prion reduction was observed for compound 5. To show that the pronounced prion clearance was not due to the binding to PrP, we performed an RT-QuIC assay showing that compound 5 is not able to halt PrP^C^ to PrP^Sc^ conversion and further aggregation. Furthermore, we showed the ability of compound 5 to inhibit the complex formation between SerpinA3n and chymotrypsin, one of its known target proteases. Although more work is needed to corroborate these early findings, the determination of dissociation constant in the micromolar range confirms the direct binding of 5 to SerpinA3n and the proposed mechanism of anti-prion action. The marked reduction of PrP^Sc^ in compound 5-treated cells infected with two different mouse-adapted scrapie strains suggests a prion-strain independent efficacy of our lead compound. Our results show the benefit of a non-PrP-directed therapeutic strategy that can help pave the way for the development of new anti-prion therapeutics, circumventing the phenomenon of prion strain resistance. We have also shown a delayed prion accumulation in *de novo* RML infected GT1 cells, treated with compound 5, compared to the untreated *de novo* infected cells. Based on these data, we suggest that compound 5 is able both to clear prions from established chronically infected cell lines and to slow down the prion accumulation process. In this scenario, with the improvement of more sensitive diagnostic tools for prion diseases,^37,38^ compound 5 could be used at the early stage of the disease to delay symptoms appearance and increase patients’ life expectancy.

However, despite the encouraging and promising *in vitro* results, the pharmacokinetic profile of compound 5 revealed low brain concentrations and high plasma clearance. Further medicinal chemistry modifications on the compound 5 scaffold could increase its brain concentration and stability and, possibly, its anti-prion efficacy. Enhanced bioavailability of compound 5 analogues could allow their use in a dual synergistic strategy with other promising PrP-targeted anti-prion therapeutics.^39^

According to the most intriguing hypothesis, serine protease inhibitors, such as SERPINA3/SerpinA3n, could bind and block the activity of protease(s) involved among other processes in the clearance of prion protein aggregates. Therefore, small molecules, such as compound 5, able to inhibit serpins activity would set the protease(s) free to further reduce prion accumulation.

It is noteworthy that SERPINA3 overexpression appears to be correlated also with Alzheimer’s disease,^12,13,40−43^ multiple system atrophy,^44^ and amyotrophic lateral sclerosis pathology;^45^ therefore, our therapeutic strategy could be potentially applied to treat other prion-like neurodegenerative disorders.

## Experimental Section

### SBVS and Similarity Search

The X-ray structure of SERPINA3 (PDB: 1AS4)^46^ was used as a receptor for our SBVS campaign. The protein was prepared using the protein preparation wizard protocol implemented in Maestro.^41^ Hydrogens were added, and charges and protonation states were assigned titrating the protein at pH 7. Short minimization steps were performed to relieve the steric clashes. The grid, used for subsequent docking calculations, was centered on a pocket at the interface of β-sheets B and C and α-helix H. Such a pocket, called the sB/sC pocket, has been identified for plasminogen activator inhibitor 1 (PDB: 4G8O), and it seemed to be conserved also for other serpins.^16^ An in-house collection of ∼15,000 nonredundant and diverse drug-like molecules was employed as a virtual library. The ligands were prepared using the LigPrep tool, implemented in Maestro. Hydrogens were added, and ionization states were generated at pH 7.4 ± 0.5. The library was filtered to retain only the molecules that obey Lipinsky’s rules and that do not bear re-active functional groups.^47^ The SBVS was performed through Glide software,^48^ using Single Precision and retaining one pose for each ligand. After a visual inspection of the best-scored poses, a first set of compounds (i.e., A–U) was selected for biological assay. Finally, based on the common structural features of the most active compounds (i.e., G and H), a similarity search was performed on the virtual library using Canvas.^49^ A second set of compounds (i.e., 1–8), bearing the 5-aminopyrazole scaffold, was selected for biological assays. The Schrödinger suite version 2015-4 was used for our calculations.

### Compound Purity Assessment

All the tested compounds were >95% pure. Compound 5 displayed ≥99% purity as determined by UPLC/MS analysis (Supporting Information Purity of compound 5). 10 mM stock solution of Compound 5 was prepared in DMSO-*d*_6_ and further diluted 20-fold with CH_3_CN–H_2_O (1:1) for analysis. The QC analyses were performed on a Waters ACQUITY UPLC/MS system consisting of a SQD (Single Quadrupole Detector) Mass Spectrometer equipped with an electrospray ionization interface and a photodiode array detector. Electrospray ionization in positive and negative modes was applied in the mass scan range 100–500 Da. The PDA range was 210–400 nm. The analyses were run on an ACQUITY UPLC BEH C18 column (100 × 2.1 mm ID, particle size 1.7 μm) with a VanGuard BEH C18 pre-column (5 × 2.1 mm ID, particle size 1.7 μm). The mobile phase was 10 mM NH_4_OAc in H_2_O at pH 5 adjusted with AcOH (A) and 10 mM NH_4_OAc in CH_3_CN–H_2_O (95:5) at pH 5 (B) with 0.5 mL/min as flow rate. A linear gradient was applied: 0–0.2 min: 10% B, 0.2–6.2 min: 10–90% B, 6.2–6.3 min: 90–100%, 6.3–7.0 min: 100% B.

### Cell Culture

Mouse neuroblastoma cell lines, either non-infected (N2a) or chronically infected with the Rocky Mountain Laboratory (RML) or 22L prion strain (ScN2a RML and ScN2a 22L, respectively), were grown in minimal essential medium −1% L-glutamax complemented with 10% fetal bovine serum (FBS), 1% non-essential amino acids, and 1% penicillin–streptomycin. Immortalized mouse hypothalamic neurons (GT1) and chronically infected GT1 cells with both RML and 22L prion strains (ScGT1 RML and ScGT1 22L, respectively) were grown in Dulbecco’s modified Eagle’s medium—1% Gluta-MAX supplemented with 10% FBS and 1% penicillin–streptomycin. All cell lines were cultivated in 10 cm^2^ Petri dishes at 37 °C under 5% CO_2_.

### Cell Treatment

Compounds from library A-U were dissolved in 100% ethanol (EtOH) and compounds from library 1–8 in dimethyl sulfoxide (DMSO) in a 200 mM stock solution. Intermediate dilutions were prepared from stock solutions. For the cell treatment, intermediate solutions were further diluted in phosphate buffer solution (PBS) 1X to a final concentration of 10 mM. Each compound was then diluted in cell culture medium to reach a final concentration of 20 or 40 μM. For the acute treatment, cells were treated with only one dose (20 or 40 μM) and then left to incubate for 4 days, whereas for the chronic treatment, they were treated with a single dose (20 μM) every week for 1 month. Mock controls were treated with vehicle only, under the same conditions.

### Assessment of Cell Viability with MTT Assay

Both un-infected and chronically infected N2a and GT1 cells, infected with RML or 22L prion strain, were maintained in culture and grown to 80% confluency. The medium was changed, and the cells were detached. Cell density was determined by cell counting using ScepterTM 2.0 Cell Counter (Millipore) and adjusted to 1 × 10^4^ cell/mL with MEM (N2a, ScN2a RML, and ScN2a 22L) or 2 × 104 with DMEM (GT1, ScGT1 RML, and ScGT1 22L). Cell suspension was added to each well of a 96-well, tissue culture-treated, clear bottom plate (Costar), and cells were allowed to settle for 1 day at 37 °C under 5% CO_2_ before the treatment with compounds. Each compound was diluted in the cell medium to the desired final concentration. After 24 h, the cell culture medium was removed and replaced by compound-containing medium. The plate was incubated at 37 °C under 5% CO_2_ for 6 days. The Thiazolyl Blue Tetrazolium Bromide (MTT, Sigma-Aldrich) was diluted in PBS 1X to a working dilution of 5 mg/mL. Cells were incubated with 20 μL of MTT solution for 3 h at 37 °C. After incubation, 100 μL of 1:1 DMSO/2-propanol solution was added to each well and the plate was kept at room temperature for 5 min before reading. The emission intensity was quantified using the EnSpire Multimode Plate Reader (PerkinElmer).

### RNA Extraction and Reverse Transcription-Quantitative Polymerase Chain Reaction (RT-qPCR)

After removing the medium, un-infected and chronically prion-infected N2a and GT1 cell lines were washed in PBS 1X and pelleted at 190*g* for 5 min. Cell pellets were resuspended in 1ml TRIzol reagent (Ambion, Life Technologies) following the manufacturer’s instructions and stored at −80 °C until further processing. Total RNA was extracted with PureLink RNA Mini Kit (Life Technologies), and on-column DNA digestion was performed using PureLink DNase Set (Life Technologies). RNA was checked for concentration and purity on a NanoDrop 2000 spectrophotometer (Thermo Scientific). cDNA was obtained starting from 3 μg of total RNA with 50 μM Oligo(dT)20, 10 mM dNTP mix, 5X First-Strand Buffer, 0.1 M DTT, 40 U RNAse inhibitor, and 200 U SuperScript III Reverse Transcriptase (Life Technologies). A negative control was performed for each sample, omitting the reverse transcriptase. Gene expression assays were performed using qPCR primer sequences (*SerpinA3n*, *Gapdh*, *Tubb3* and *Actb*) and the protocols reported in Vanni et al. 2017.^12^ RT-qPCR analysis was performed using five samples for each condition of un-infected and prion-infected N2a and GT1 cells. The relative expression ratio (fold change, FC) was calculated using the 2^–ΔΔCT^ method.^50^ Briefly, ΔC_T_ was calculated subtracting the C_T_ of the reference genes from the C_T_ of *SerpinA3n*, both for “test” (either RML or 22L prion-infected cell) and “calibrator” (un-infected cells). Then, ΔΔC_T_ was obtained subtracting the mean ΔC_T_ of the population of calibrator samples (five samples for both uninfected N2a and GT1 cells) was subtracted from the ΔC_T_ of each sample. Fold change values smaller than 1 were converted using the equation −1/FC, for representation.

### Collection of Conditioned Media, Cell Lysis, and Proteinase K (PK) Digestion

For extracellular SerpinA3n detection conditioned media (CM) of un-infected and prion-infected N2a and GT1 cell lines were collected, cleared, and concentrated following the protocol reported by Gueugneau et al. in 2018.^24^ Briefly, after 24 h incubation in serum-free medium, the CM was centrifuged for 10 min at 300*g*, and for another 20 min at 2000*g*, to discard cell debris. CM was subsequently concentrated using the Amicon Ultra-4 30kDa cut-off spin Column (Millipore, Watford, UK). For intracellular SerpinA3n and PrP detection, after removing the medium, cells were washed with PBS 1X and lysed on ice in lysis buffer (10 mM Tris–HCl pH 8.0, 150 mM NaCl, 0.5% Nonidet P-40, 0.5% deoxycholic acid sodium salt). Nuclei and large debris were removed by centrifugation at 2000 rpm for 10 min at 4 °C in a bench microfuge (Eppendorf). The protein concentration of cleared cell lysate and conditioned media was determined using the bicinchoninic acid protein quantification kit (Thermo Fisher Scientific). For intracellular and extracellular SerpinA3n detection, 100 μg of cell lysate and 50 μg of conditioned medium, respectively, were added into a 5X SDS-PAGE loading buffer in a 1:4 ratio. For PrP detection, cell lysates were split into two parts. One part (250 μg) was treated with 5 μg of PK (Roche Diagnostics Corp.) at 37 °C for 1 h. The reaction was arrested with 2 mM of phenylmethylsulphonyl fluoride (PMSF, Sigma-Aldrich). The PK-digested samples were precipitated by centrifugation at 186,000*g* for 75 min at 4 °C in an ultracentrifuge (Beckman Coulter) and the pellet was resuspended in 1X SDS- PAGE loading buffer. The non-PK-digested samples (25 μg) were added into a 2X SDS-PAGE loading buffer in a 1:1 ratio. All samples were boiled for 10 min at 100 °C. All samples were stored at −20 °C until further processing or analysis.

### Western Blotting

Samples were loaded onto a 10% (for SerpinA3n detection) or 12% (for PrP detection) Acrylamide/Bis-acrylamide (Sigma-Aldrich) gels, separated by SDS-PAGE, and transferred to the PVDF membrane. The membranes were blocked with 5% non-fat milk in TBST (Tris 200 mM, NaCl 1.5 mM, 1% Tween-20, Sigma-Aldrich). For extracellular SerpinA3n, Ponceau S staining of the membrane, before milk blocking, was performed to verify the accuracy of sample loading. For SerpinA3n detection, membranes were incubated with the polyclonal mouse SerpinA3n antibody (1:500 R&D Systems) followed by the rabbit anti-goat HRP secondary anti-body (1:1000). For PrP detection, anti-PrP Fab W226 (kindly provided by Prof. Krammerer) antibody was used, followed by the goat anti-mouse HRP secondary antibody (1:1000). Monoclonal anti-β-actin-peroxidase antibody (1:10,000 Sigma-Aldrich) was used to normalize intracellular SerpinA3n and total PrP signals, from the non-PK- digested samples. The membranes were visualized by chemiluminescence using Amersham ECL Prime (GE Healthcare). Densitometric analysis was carried out using UVIBand software.

### Production and Purification of Recombinant SerpinA3n

SerpinA3n recombinant protein was produced as already described with some modifications.^51^ A pET(11a) expression vector containing the C-terminally (6×) His-tagged murine SerpinA3n (Genetech) was used to transform *Escherichia coli* BL21 (DE3) pLysS cells. Cells were grown in Luria–Bertani medium at 25 °C in the presence of ampicillin (100 μg/mL) until OD600 = 1; when protein production was induced with 0.1 mM isopropyl 1-thio-d-galactopyranoside, the growth temperature was lowered to 15 °C. After 21 h of induction, bacteria were pelleted through centrifugation (9000*g* for 10 min at 4 °C) and the supernatant was discarded. The pellet was washed in 0.9% physiological solution and collected after centrifugation (9000*g* for 10 min at 4 °C). Around 12 g of the pellet was then kept at −80 °C until purification was carried out. The frozen pellet was resuspended in 25 mL (per cell paste) of buffer A (50 mM Tris–HCl, pH 8.0, 300 mM NaCl, 20 mM Imidazole), to which one tab of cOmplete Protease Inhibitor Cocktail (Roche), 250 μL of 0.2 M PMSF, and 125 μL of 25 μg/mL DNase were added (homogenization buffer). Bacterial cell lysis was performed by sonication using 5 cycles of 60 s on and 60 s of rest in ice. Cell debris was discarded after 1 h of centrifugation (12,000*g* at 4 °C), and the supernatant was resuspended in 25 mL of homogenization buffer. Crude extract was loaded onto the HisTrap Fast Flow Crude column (GE Healthcare). The purification was performed with ÄKTA pure chromatography keeping the system at 4 °C. After a washing step in 50 mM Tris–HCl, pH 8.0, 300 mM NaCl, 20 mM imidazole, and 1 mM PMSF to elute all proteins that do not bind to the column, the protein of interest was eluted by applying a linear imidazole gradient from 20 up to 500 mM imidazole. Protein expression and purity of eluted fractions were checked with SDS-PAGE followed by Coomassie brilliant blue staining. The fractions containing the higher amount of purified SerpinA3n were pooled together, dialyzed in 10 mM Tris–HCl, 50 mM KCl pH 8.0, and concentrated using Amicon Ultra-15 Centrifugal Filters 30 kDa cutoff (Merk Millipore).

### Dose–Response Curves for EC_50_ Calculations

For EC_50_ (concentration responsible for 50% of prion accumulation decrease) assessment, both N2a and GT1 prion-infected (with RML and 22L strains) cell lines were treated for 3 days with compound 5 in a concentration range from 1 to 50 μM. Dose–response curves and relative EC_50_ were generated as log concentration of compound 5 versus percentage of PrP^Sc^ amount in treated cells (from Western Blot analysis) using GraphPad Prism 7.0 software (*n* = 3).

### Dose–Survival Curves for LD_50_ Calculations

For LD_50_ (concentration leading to 50% of cell viability) both N2a and GT1 prion-infected (with RML and 22L strains) cell lines were treated for 6 days with compound 5 in a concentration range from 10 to 100 μM. Dose–survival curves and relative LD_50_ were generated as log concentration of compound 5 versus percentage of cell viability (from MTT assay) using GraphPad Prism 7.0 software (*n* = 3).

### Complex Formation Assay

8 μM recombinant SerpinA3n was incubated with 2 μM bovine α-chymotrypsin (Merk) and with increasing concentrations of Compound 5 (from 10 nM to 1 mM) or vehicle (DMSO) for 30 min at 37 °C with shaking in the assay buffer (10 mM Tris–HCl, 50 mM KCl, pH 8.0).^52^ Samples were separated by Acrylamide/Bis-acrylamide (Sigma-Aldrich) 10% SDS-PAGE and stained with Coomassie brilliant blue.

### Isothermal Titration Calorimetry

ITC experiments were performed using a Malvern PEAQ-ITC microcalorimeter, at 25 °C, with a 270 μL sample cell and a computer-controlled microsyringe for titrant injections. SerpinA3n (16 μM) and 5 (160 μM) samples were in 25 mM Tris, 50 mM KCl, 0.3% DMF, pH 8.0. After baseline stabilization, a further delay of 60 s was used before the first injection. In each individual titration, starting 5 injections of 0.4 μL in 0.8 s were followed by other 12 injections of 3 μL with a duration of 6 s each. A delay of 150 s was applied between each c5 injection. The Wiseman plot of integrated data was automatically obtained by software analysis, and then it was fitted by a theoretical binding curve using the one-site model.

### *De Novo* Prion Infection of GT1 Cell Lines

ScGT1 RML cells were grown to confluence and then sonicated. The resulted lysates (coming from three 100% confluent 10 cm^2^ Petri dishes) were added to the medium of GT1 cells, at 10–20% of confluence. The medium was refreshed 3 days after infection and, from the 7th day, cells were split four times, and at each passage, cells were lysed to be tested for PrP^Sc^ presence.

### Real-Time Quaking-Induced Conversion (RT-QuIC) Assay

Recombinant full-length mouse PrP (MoPrP) (23–231) production and purification were performed as previously described.^21^ After purification, aliquots of MoPrP were stored at −80 °C in 10 mM phosphate buffer (pH 5.8). Before each test, the protein solution was allowed to thaw at room temperature and filtered using Millex-GV filter 0.22 μm (Millipore). The final reaction volume was 100 μL loaded into the plate (ViewPlate-96 F TC/50 × 1B, Perkin Elmer), and the reagents (Sigma-Aldrich) were concentrated as follows: 150 mM NaCl, 0.002% SDS, 1X PBS, 1 mM EDTA, 10 μM ThT and 0.2 mg FLMoPrP mL^–1^. The seed consisted of sonicated and phosphotungstic acid-precipitated ScN2a-RML cell lysates. Before the sonication, the cells were collected in 100 μl of PBS 1X; after the sonication, the sample was quantified using the BCA assay, to use it as a seed (1 μg of the protein). After the addition of 10 μL of the seed, the plate was sealed with a sealing film (PerkinElmer) and inserted into a FLUOstar OPTIMA microplate reader (BMG Labtech). The plate was shaken for 1 min at 600 rpm (double orbital) and incubated for 1 min at 45 °C. Fluorescence readings (480 nm) were taken every 30 min (30 ashes per well at 450 nm). Given the rapid response, a specific threshold was set to decrease the likelihood of false positives. A sample was considered positive if the mean of the highest two fluorescence values (AU) of the replicates was higher than 10,000 AU and at least two out of three replicates crossed the threshold that was set at 30 h. This reaction cutoff was established because in all the experiments there were wells only with full-length MoPrP (23–231), and in these cases, no positive RT-QuIC reactions were observed until after 30 h.

### Immunofluorescence of Fixed Cells

N2a cells were seeded to semi-confluence in each well of a 24-well plate containing the coverslips and an appropriate culture medium for 24 h. After 1 day of incubation, only for surface staining of the PrP^C^, cells were put on ice for 15 min and stained with the primary antibody (W226 1:500) in culture medium for 20 min. The medium was removed, and the cells were washed twice with PBS 1X. The cells were then fixed using 4% of paraformaldehyde (PFA) for 20 min. PFA was removed, and the cells were washed 3 times with PBS 1X. Blocking buffer, consisting of 1% bovine serum albumin (BSA) in PBS 1X, was added to the cells for 1 h at room temperature. After the blocking, the cells were incubated with a secondary antibody (Goat anti-mouse-AlexaFluor488, Life Technologies) diluted 1:200 in the incubation buffer (1% BSA in PBS 1X) for 1 h at room temperature in the dark. For Total PrP staining, after 24 h from seeding, the cells were fixed in 4% of PFA for 20 min. PFA was removed, and the cells were washed three times with PBS 1X. Cells were then permeabilized with 0.02% of TritonX-100 in PBS 1X for 5 min and washed again three times with PBS 1X. Cells were then blocked in 1% BSA for 1 h at room temperature and incubated with primary antibody (W226 1:500) in incubation buffer (1% BSA in PBS 1X) for 1 h at room temperature. Cells were washed three times within 1% BSA and then incubated with the secondary antibody (Goat anti-mouse-AlexaFluor488, Life Technologies) diluted 1:200 in the incubation buffer (1% BSA in PBS 1X) for 1 h at room temperature in the dark. For both stainings, cells were washed three times in PBS 1X and then the coverslips were mounted with a drop of Fluoromount-G (Invitrogen). The coverslips were sealed with nail polish to prevent drying and movement under the microscope. Images were acquired with a C1 confocal microscope (Nikon). An FITC filter was used for the detection of PrP-specific staining.

### Isobologram Analysis

ScN2a RML cells were treated with compound 5 and quinacrine, in a concentration range from 10–2 μM and 1–0.2 μM, respectively. Dose–response curves and isobologram plots were generated in GraphPad Prism 7.0 software. The theoretical EC_50,add_ value (assuming an additive interaction between compound 5 and quinacrine) was calculated using eqs 1 and 2, as follows
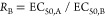
1
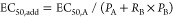
2where *R*_B_ represents the potency ratio of drugs A and B, EC_50,A_ and EC_50,B_ represent the half maximal effective concentrations of drugs A and B (respectively) when used alone in a treatment. *P*_A_ and *P*_B_ are the proportions of drugs A and B used in a combination treatment.

The dose gradient of compound 5 and quinacrine was set and the measured EC_50_, mix value (the dose of each compound that achieves 50% efficacy, when used in a combination) was determined from the dose–response data. To evaluate drug interaction, the interaction index (γ) was calculated using the eq 3, as follows
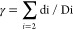
3where “di” represents the dose required by each single drug, used in a combination treatment, to achieve EC_50_ and “Di” represents the dose required by each drug to achieve EC_50_, when used alone.^53^

### Animal Models

Male C57BL/6 mice, weighing 22–24 g, were used (Charles River). All procedures were performed following the Ethical Guidelines of the European Communities Council (Directive 2010/63/EU of 22 September 2010) and accepted by the Italian Ministry of Health. All efforts were made to minimize animal suffering and to use the minimal number of animals required to produce reliable results, according to the “3Rs concept”. Animals were group-housed in ventilated cages and had free access to food and water. They were maintained under a 12 h light/dark cycle (lights on at 8:00 am) at controlled temperature (21 ± 1 °C) and relative humidity (55 ± 10%).

### Pharmacokinetic *in vivo* Studies

Compound 5 was administered orally (P.O.) and intravenously (I.V.) to C57BL/6 male mice at 10 and 3 mg/kg. Vehicle was: PEG400/Tween 80/Saline solution at 10/10/80% in volume respectively. Three animals per time point were treated. Blood samples and brains at 0, 5, 15, 30, 60, 120, and 240 min after administration were collected for both P.O. and I.V. arms. Plasma was separated from blood by centrifugation for 15 min at 1,500 rpm at 4 °C, collected in an Eppendorf tube, and frozen (−80 °C). Brain samples were homogenized in PBS 1X and were then split in two aliquots kept at −80 °C until analysis. An aliquot was used for compound brain level evaluations, following the same procedure described below for plasma samples. The second aliquot was kept for protein content evaluation by BCA. Control animals treated with vehicle only were also included in the experimental protocol.

### Pharmacokinetic Measurements

Plasma samples were centrifuged at 21.100*g* for 15 min at 4 °C, while homogenized brain samples were vigorously whirled. An aliquot of each sample was extracted (1:3) with cold CH_3_CN containing 200 nM of an appropriate internal standard. A calibration curve was prepared in both naïve mouse plasma and naïve mouse brain homogenate over a 1 nM to 10 μM range. Three quality control samples were prepared by spiking the parent compound in both naïve mouse plasma and naïve brain homogenate to 20, 200, and 2000 nM as final concentrations. The calibrators and quality control samples were extracted (1:3) with the same extraction solution as the plasma and brain samples. The plasma and brain samples, calibrators, and quality control samples were centrifuged at 3.270*g* for 15 min at 4 °C. The supernatants were further diluted (1:1) with H_2_O and analyzed by LC–MS/MS on a Waters ACQUITY UPLC/MS TQD system consisting of a Triple Quadrupole Detector Mass Spectrometer equipped with an Electrospray Ionization interface and a Photodiode Array eλ Detector from Waters Inc. (Milford, MA, USA). Electrospray ionization was applied in positive mode. Compound-dependent parameters such as MRM transitions and collision energy were developed for the parent compound and the internal standard. The analyses were run on an ACQUITY UPLC BEH C18 (50 × 1 mm ID, particle size 1.7 μm) with a KrudKatcher ULTRA HPLC In-Line Filter (0.5 μm × 0.004 in. ID) at 40 °C, using H_2_O + 0.1% HCOOH (A) and CH_3_CN + 0.1% HCOOH (B) as mobile phase at 0.1 mL/min. A linear gradient was applied starting at 10% B with an initial hold for 0.5 min, then 10–100% B in 3 min, followed by a hold for 0.5 min at 100% B. All samples (plasma and brain samples, calibrators, and quality controls) were quantified by MRM peak area response factor to determine the levels of the parent compound in plasma and brain. The plasma concentrations versus time were plotted, and the profiles were fitted using PK Solutions Excel Application (Summit Research Service, USA) to determine the pharmacokinetic parameters.

### Statistical Analysis

Statistical analysis was performed using GraphPad Prism 7.0 software. The normal distribution of data was assessed by the D’Agostino–Pearson normality test. For RT-qPCR analysis, differences between the ΔCTs of prion-infected and uninfected cells were assessed with the Kruskal–Wallis test for not normally distributed data. The level of significance was calculated using Dunn’s multiple comparisons test between ΔCTs of prion-infected and uninfected cells. β-actin normalized Western blot signal values obtained from control and treated cells were normalized to the mean of the control samples for each experiment (in technical triples). Groups were compared by using the non-parametric Friedman test and Wilcoxon’s matched pairs ranked test or Kruskal–Wallis and Dunn’s multiple comparisons test. *P*-values ≤ 0.05 were considered statistically significant.

## Supporting Information Available

Compounds, SerpinA3n expression in uninfected and prion-infected GT1 and N2a cell lines, cell viability assay of tested molecules, anti-prion effect of first and second library molecules, binding model of compound 5, compound 5 inhibitory activity on SerpinA3n-chymotrypsin complex formation, ITC measurements, dose–survival curves of compound 5 on RML- and 22L-infected GT1 and N2a cell lines, d*e novo* infection with compound 5, cell viability assay and PrP^C^ amount in compound 5-treated un-infected cells and PrP^C^ aggregation propensity in presence of compound 5, PrP^C^ localization upon compound 5 treatment, and purity of compound 5 (PDF)
SMILES molecular formula strings (CSV)
Molecular docking of compound 5 and SerpinA3n (PDB)

## Supplementary Material

jm2c00205_si_002.csv

jm2c00205_si_003.pdb

## Abbreviations

BSA: bovine serum albumin
BSE: bovine spongiform encephalopathies
CM: conditioned media
DMSO: dimethyl sulfoxide
EC_50_: concentration responsible for 50% of prion accumulation decrease
EtOH: ethanol
FBS: fetal bovine serum
GT1: mouse hypothalamic neurons
ITC: isothermal titration calorimetry
LD_50_: concentration leading to 50% of cell viability
MTT: thiazolyl blue tetrazolium bromide
N2a: mouse neuroblastoma cell
PBS: phosphate buffer solution
PDB: protein data bank
PFA: paraformaldehyde
PK: proteinase K
PrP^C^: cellular prion protein
PrP^Sc^: scrapie prion protein
PVDF: polyvinylidene fluoride
RML: rocky mountain laboratory
RT-qPCR: reverse transcription-quantitative polymerase chain reaction
RT-QuIC: real-time quaking-induced conversion
SBVS: structure-based virtual screening
TBS-T: tris-buffered saline-tween
TSEs: transmissible spongiform encephalopathies

## References

[ref1] Scheckel C.; Aguzzi A. Prions, prionoids and protein misfolding disorders. Nat. Rev. Genet. 2018, 19, 405–418. 10.1038/s41576-018-0011-4. 29713012

[ref2] Prusiner S. B. Prions. Proc. Natl. Acad. Sci. U.S.A. 1998, 95, 13363–13383. 10.1073/pnas.95.23.13363. 9811807 PMC33918

[ref3] Legname G. Elucidating the function of the prion protein. PLoS Pathog. 2017, 13, e1006458 10.1371/journal.ppat.1006458. 28859171 PMC5578479

[ref4] Jucker M.; Walker L. C. Propagation and spread of pathogenic protein assemblies in neurodegenerative diseases. Nat. Neurosci. 2018, 21, 1341–1349. 10.1038/s41593-018-0238-6. 30258241 PMC6375686

[ref5] Aguzzi A.; Calella A. M. Prions: protein aggregation and infectious diseases. Physiol. Rev. 2009, 89, 1105–1152. 10.1152/physrev.00006.2009. 19789378

[ref6] Colini Baldeschi A.; Vanni S.; Zattoni M.; Legname G. Novel regulators of PrP(C) expression as potential therapeutic targets in prion diseases. Expert Opin. Ther. Targets 2020, 24, 759–776. 10.1080/14728222.2020.1782384. 32631090

[ref7] Kuwata K.; Nishida N.; Matsumoto T.; Kamatari Y. O.; Hosokawa-Muto J.; Kodama K.; Nakamura H. K.; Kimura K.; Kawasaki M.; Takakura Y.; Shirabe S.; Takata J.; Kataoka Y.; Katamine S. Hot spots in prion protein for pathogenic conversion. Proc. Natl. Acad. Sci. U.S.A. 2007, 104, 11921–11926. 10.1073/pnas.0702671104. 17616582 PMC1924567

[ref8] Spagnolli G.; Massignan T.; Astolfi A.; Biggi S.; Rigoli M.; Brunelli P.; Libergoli M.; Ianeselli A.; Orioli S.; Boldrini A.; Terruzzi L.; Bonaldo V.; Maietta G.; Lorenzo N. L.; Fernandez L. C.; Codeseira Y. B.; Tosatto L.; Linsenmeier L.; Vignoli B.; Petris G.; Gasparotto D.; Pennuto M.; Guella G.; Canossa M.; Altmeppen H. C.; Lolli G.; Biressi S.; Pastor M. M.; Requena J. R.; Mancini I.; Barreca M. L.; Faccioli P.; Biasini E. Pharmacological inactivation of the prion protein by targeting a folding intermediate. Commun. Biol. 2021, 4, 62 10.1038/s42003-020-01585-x. 33437023 PMC7804251

[ref9] Raymond G. J.; Zhao H. T.; Race B.; Raymond L. D.; Williams K.; Swayze E. E.; Graffam S.; Le J.; Caron T.; Stathopoulos J.; O’Keefe R.; Lubke L. L.; Reidenbach A. G.; Kraus A.; Schreiber S. L.; Mazur C.; Cabin D. E.; Carroll J. B.; Minikel E. V.; Kordasiewicz H.; Caughey B.; Vallabh S. M. Antisense oligonucleotides extend survival of prion-infected mice. JCI Insight 2019, 4, 16 10.1172/jci.insight.131175. PMC6777807 31361599

[ref10] Minikel E. V.; Zhao H. T.; Le J.; O’Moore J.; Pitstick R.; Graffam S.; Carlson G. A.; Kavanaugh M. P.; Kriz J.; Kim J. B.; Ma J.; Wille H.; Aiken J.; McKenzie D.; Doh-Ura K.; Beck M.; O’Keefe R.; Stathopoulos J.; Caron T.; Schreiber S. L.; Carroll J. B.; Kordasiewicz H. B.; Cabin D. E.; Vallabh S. M. Prion protein lowering is a disease-modifying therapy across prion disease stages, strains and endpoints. Nucleic Acids Res. 2020, 48, 10615–10631. 10.1093/nar/gkaa616. 32776089 PMC7641729

[ref11] Barbisin M.; Vanni S.; Schmädicke A.-C.; Montag J.; Motzkus D.; Opitz L.; Salinas-Riester G.; Legname G. Gene expression profiling of brains from bovine spongiform encephalopathy (BSE)-infected cynomolgus macaques. BMC Genom. 2014, 15, 434 10.1186/1471-2164-15-434. PMC4061447 24898206

[ref12] Vanni S.; Moda F.; Zattoni M.; Bistaffa E.; De Cecco E.; Rossi M.; Giaccone G.; Tagliavini F.; Haïk S.; Deslys J. P.; Zanusso G.; Ironside J. W.; Ferrer I.; Kovacs G. G.; Legname G. Differential overexpression of SERPINA3 in human prion diseases. Sci. Rep. 2017, 7, 15637 10.1038/s41598-017-15778-8. 29142239 PMC5688139

[ref13] Zattoni M.; Mearelli M.; Vanni S.; Colini Baldeschi A.; Tran T. H.; Ferracin C.; Catania M.; Moda F.; Di Fede G.; Giaccone G.; Tagliavini F.; Zanusso G.; Ironside J. W.; Ferrer I.; Legname G. Serpin Signatures in Prion and Alzheimer’s Diseases. Mol. Neurobiol. 2022, 59, 3778 10.1007/s12035-022-02817-3. 35416570 PMC9148297

[ref14] Kellici T. F.; Pilka E. S.; Bodkin M. J. Small-molecule modulators of serine protease inhibitor proteins (serpins). Drug Discov. Today 2021, 26, 442–454. 10.1016/j.drudis.2020.11.012. 33259801

[ref15] Savardi A.; Borgogno M.; Narducci R.; La Sala G.; Ortega J. A.; Summa M.; Armirotti A.; Bertorelli R.; Contestabile A.; De Vivo M.; Cancedda L. Discovery of a Small Molecule Drug Candidate for Selective NKCC1 Inhibition in Brain Disorders. Chem 2020, 6, 2073–2096. 10.1016/j.chempr.2020.06.017. 32818158 PMC7427514

[ref16] Li S. H.; Reinke A. A.; Sanders K. L.; Emal C. D.; Whisstock J. C.; Stuckey J. A.; Lawrence D. A. Mechanistic characterization and crystal structure of a small molecule inactivator bound to plasminogen activator inhibitor-1. Proc. Natl. Acad. Sci. U.S.A. 2013, 110, E4941–E4949. 10.1073/pnas.1216499110. 24297881 PMC3870673

[ref17] Schätzl H. M.; Laszlo L.; Holtzman D. M.; Tatzelt J.; DeArmond S. J.; Weiner R. I.; Mobley W. C.; Prusiner S. B. A hypothalamic neuronal cell line persistently infected with scrapie prions exhibits apoptosis. J. Virol. 1997, 71, 8821–8831. 10.1128/jvi.71.11.8821-8831.1997. 9343242 PMC192348

[ref18] Butler D. A.; Scott M. R.; Bockman J. M.; Borchelt D. R.; Taraboulos A.; Hsiao K. K.; Kingsbury D. T.; Prusiner S. B. Scrapie-infected murine neuroblastoma cells produce protease-resistant prion proteins. J. Virol. 1988, 62, 1558–1564. 10.1128/jvi.62.5.1558-1564.1988. 3282080 PMC253182

[ref19] Krance S. H.; Luke R.; Shenouda M.; Israwi A. R.; Colpitts S. J.; Darwish L.; Strauss M.; Watts J. C. Cellular models for discovering prion disease therapeutics: Progress and challenges. J. Neurochem. 2020, 153, 150–172. 10.1111/jnc.14956. 31943194

[ref20] Altieri A.; Spiridonov E. A.; Sivtzev S. I.; Ishibashi D.; Biggi S.; Nishida N.; Biasini E.; Kurkin A. V. Generation, optimization and characterization of novel anti-prion compounds. Bioorg. Med. Chem. 2020, 28, 115717 10.1016/j.bmc.2020.115717. 33065443

[ref21] Zaccagnini L.; Rossetti G.; Tran T. H.; Salzano G.; Gandini A.; Colini Baldeschi A.; Bolognesi M. L.; Carloni P.; Legname G. In silico/in vitro screening and hit evaluation identified new phenothiazine anti-prion derivatives. Eur. J. Med. Chem. 2020, 196, 112295 10.1016/j.ejmech.2020.112295. 32325366

[ref22] Ghaemmaghami S.; Russo M.; Renslo A. R. Successes and challenges in phenotype-based lead discovery for prion diseases. J. Med. Chem. 2014, 57, 6919–6929. 10.1021/jm5001425. 24762293 PMC4148153

[ref23] Sergi D.; Campbell F. M.; Grant C.; Morris A. C.; Bachmair E.-M.; Koch C.; McLean F. H.; Muller A.; Hoggard N.; de Roos B.; Porteiro B.; Boekschoten M. V.; McGillicuddy F. C.; Kahn D.; Nicol P.; Benzler J.; Mayer C.-D.; Drew J. E.; Roche H. M.; Muller M.; Nogueiras R.; Dieguez C.; Tups A.; Williams L. M. SerpinA3N is a novel hypothalamic gene upregulated by a high-fat diet and leptin in mice. Genes Nutr. 2018, 13, 28 10.1186/s12263-018-0619-1. 30519364 PMC6263559

[ref24] Gueugneau M.; d’Hose D.; Barbé C.; de Barsy M.; Lause P.; Maiter D.; Bindels L. B.; Delzenne N. M.; Schaeffer L.; Gangloff Y.-G.; Chambon C.; Coudy-Gandilhon C.; Béchet D.; Thissen J.-P. Increased Serpina3n release into circulation during glucocorticoid-mediated muscle atrophy. J. Cachexia Sarcopenia Muscle 2018, 9, 929–946. 10.1002/jcsm.12315. 29989354 PMC6204594

[ref25] Law R. H.; Zhang Q.; McGowan S.; Buckle A. M.; Silverman G. A.; Wong W.; Rosado C. J.; Langendorf C. G.; Pike R. N.; Bird P. I.; Whisstock J. C. An overview of the serpin superfamily. Genome Biol. 2006, 7, 216 10.1186/gb-2006-7-5-216. 16737556 PMC1779521

[ref26] Horvath A. J.; Irving J. A.; Rossjohn J.; Law R. H.; Bottomley S. P.; Quinsey N. S.; Pike R. N.; Coughlin P. B.; Whisstock J. C. The murine orthologue of human antichymotrypsin: a structural paradigm for clade A3 serpins. J. Biol. Chem. 2005, 280, 43168–43178. 10.1074/jbc.m505598200. 16141197

[ref27] Wilham J. M.; Orrú C. D.; Bessen R. A.; Atarashi R.; Sano K.; Race B.; Meade-White K. D.; Taubner L. M.; Timmes A.; Caughey B. Rapid end-point quantitation of prion seeding activity with sensitivity comparable to bioassays. PLoS Pathog. 2010, 6, e1001217 10.1371/journal.ppat.1001217. 21152012 PMC2996325

[ref28] Korth C.; May B. C. H.; Cohen F. E.; Prusiner S. B. Acridine and phenothiazine derivatives as pharmacotherapeutics for prion disease. Proc. Natl. Acad. Sci. U.S.A. 2001, 98, 9836–9841. 10.1073/pnas.161274798. 11504948 PMC55539

[ref29] Vanni S. Omics of Prion Diseases. Prog. Mol. Biol. Transl. Sci. 2017, 150, 409–431. 10.1016/bs.pmbts.2017.05.004. 28838672

[ref30] Miele G.; Seeger H.; Marino D.; Eberhard R.; Heikenwalder M.; Stoeck K.; Basagni M.; Knight R.; Green A.; Chianini F.; Wüthrich R. P.; Hock C.; Zerr I.; Aguzzi A. Urinary alpha1-antichymotrypsin: a biomarker of prion infection. PLoS One 2008, 3, e3870 10.1371/journal.pone.0003870. 19057641 PMC2586086

[ref31] Campbell I. L.; Eddleston M.; Kemper P.; Oldstone M. B.; Hobbs M. V. Activation of cerebral cytokine gene expression and its correlation with onset of reactive astrocyte and acute-phase response gene expression in scrapie. J. Virol. 1994, 68, 2383–2387. 10.1128/jvi.68.4.2383-2387.1994. 8139024 PMC236715

[ref32] Dandoy-Dron F.; Benboudjema L.; Guillo F.; Alexandre Jaegly A.; Jasmin C.; Dormont D.; Tovey M. G.; Dron M. Enhanced levels of scrapie responsive gene mRNA in BSE-infected mouse brain. Mol. Brain Res. 2000, 76, 173–179. 10.1016/s0169-328x(00)00028-0. 10719228

[ref33] Riemer C.; Neidhold S.; Burwinkel M.; Schwarz A.; Schultz J.; Krätzschmar J.; Mönning U.; Baier M. Gene expression profiling of scrapie-infected brain tissue. Biochem. Biophys. Res. Commun. 2004, 323, 556–564. 10.1016/j.bbrc.2004.08.124. 15369787

[ref34] Xiang W.; Windl O.; Wunsch G.; Dugas M.; Kohlmann A.; Dierkes N.; Westner I. M.; Kretzschmar H. A. Identification of differentially expressed genes in scrapie-infected mouse brains by using global gene expression technology. J. Virol. 2004, 78, 11051–11060. 10.1128/jvi.78.20.11051-11060.2004. 15452225 PMC521804

[ref35] Chen C.; Xu X.-F.; Zhang R.-Q.; Ma Y.; Lv Y.; Li J.-L.; Shi Q.; Xiao K.; Sun J.; Yang X.-D.; Shi Q.; Dong X.-P. Remarkable increases of alpha1-antichymotrypsin in brain tissues of rodents during prion infection. Prion 2017, 11, 338–351. 10.1080/19336896.2017.1349590. 28956708 PMC5639854

[ref36] Moda F.; Bolognesi M. L.; Legname G. Novel screening approaches for human prion diseases drug discovery. Expet Opin. Drug Discov. 2019, 14, 983–993. 10.1080/17460441.2019.1637851. 31271065

[ref37] Orrú C. D.; Bongianni M.; Tonoli G.; Ferrari S.; Hughson A. G.; Groveman B. R.; Fiorini M.; Pocchiari M.; Monaco S.; Caughey B.; Zanusso G. A test for Creutzfeldt-Jakob disease using nasal brushings. N. Engl. J. Med. 2014, 371, 519–529. 10.1056/NEJMoa1315200. 25099576 PMC4186748

[ref38] Mammana A.; Baiardi S.; Rossi M.; Franceschini A.; Donadio V.; Capellari S.; Caughey B.; Parchi P. Detection of prions in skin punch biopsies of Creutzfeldt-Jakob disease patients. Ann. Clin. Transl. Neurol. 2020, 7, 559–564. 10.1002/acn3.51000. 32141717 PMC7187701

[ref39] Zattoni M.; Legname G. Tackling prion diseases: a review of the patent landscape. Expert Opin. Ther. Pat. 2021, 31, 1097 10.1080/13543776.2021.1945033. 34134584

[ref40] Abraham C. R.; Selkoe D. J.; Potter H. Immunochemical identification of the serine protease inhibitor alpha 1-antichymotrypsin in the brain amyloid deposits of Alzheimer’s disease. Cell 1988, 52, 487–501. 10.1016/0092-8674(88)90462-x. 3257719

[ref100] Abraham C. Reactive astrocytes and alpha1-antichymotrypsin in Alzheimer’s disease. Neurobiol. Aging 2001, 22, 931–936. 10.1016/s0197-4580(01)00302-5. 11755001

[ref42] Nilsson L. N. G.; Bales K. R.; DiCarlo G.; Gordon M. N.; Morgan D.; Paul S. M.; Potter H. Alpha-1-antichymotrypsin promotes beta-sheet amyloid plaque deposition in a transgenic mouse model of Alzheimer’s disease. J. Neurosci. 2001, 21, 1444–1451. 10.1523/jneurosci.21-05-01444.2001. 11222634 PMC6762932

[ref43] Baker C.; Belbin O.; Kalsheker N.; Morgan K. SERPINA3 (aka alpha-1-antichymotrypsin). Front. Biosci. 2007, 12, 2821–2835. 10.2741/2275. 17485262

[ref44] Mills J. D.; Ward M.; Kim W. S.; Halliday G. M.; Janitz M. Strand-specific RNA-sequencing analysis of multiple system atrophy brain transcriptome. Neuroscience 2016, 322, 234–250. 10.1016/j.neuroscience.2016.02.042. 26922980

[ref45] Sanfilippo C.; Longo A.; Lazzara F.; Cambria D.; Distefano G.; Palumbo M.; Cantarella A.; Malaguarnera L.; Di Rosa M. CHI3L1 and CHI3L2 overexpression in motor cortex and spinal cord of sALS patients. Mol. Cell. Neurosci. 2017, 85, 162–169. 10.1016/j.mcn.2017.10.001. 28989002

[ref46] Lukacs C. M.; Rubin H.; Christianson D. W. Engineering an anion-binding cavity in antichymotrypsin modulates the ″spring-loaded″ serpin-protease interaction. Biochemistry 1998, 37, 3297–3304. 10.1021/bi972359e. 9521649

[ref41] Madhavi Sastry G.; Adzhigirey M.; Day T.; Annabhimoju R.; Sherman W. Protein and ligand preparation: parameters, protocols, and influence on virtual screening enrichments. J. Comput. Aided Mol. Des. 2013, 27, 221–234. 10.1007/s10822-013-9644-8. 23579614

[ref47] Lipinski C. A. Lead- and drug-like compounds: the rule-of-five revolution. Drug Discov. Today Technol. 2004, 1, 337–341. 10.1016/j.ddtec.2004.11.007. 24981612

[ref48] Friesner R. A.; Banks J. L.; Murphy R. B.; Halgren T. A.; Klicic J. J.; Mainz D. T.; Repasky M. P.; Knoll E. H.; Shelley M.; Perry J. K.; Shaw D. E.; Francis P.; Shenkin P. S. Glide: a new approach for rapid, accurate docking and scoring. 1. Method and assessment of docking accuracy. J. Med. Chem. 2004, 47, 1739–1749. 10.1021/jm0306430. 15027865

[ref49] Duan J.; Dixon S. L.; Lowrie J. F.; Sherman W. Analysis and comparison of 2D fingerprints: insights into database screening performance using eight fingerprint methods. J. Mol. Graph. Model. 2010, 29, 157–170. 10.1016/j.jmgm.2010.05.008. 20579912

[ref50] Livak K. J.; Schmittgen T. D. Analysis of relative gene expression data using real-time quantitative PCR and the 2(-Delta Delta C(T)) Method. Methods 2001, 25, 402–408. 10.1006/meth.2001.1262. 11846609

[ref51] Visentin C.; Broggini L.; Sala B. M.; Russo R.; Barbiroli A.; Santambrogio C.; Nonnis S.; Dubnovitsky A.; Bolognesi M.; Miranda E.; Achour A.; Ricagno S. Glycosylation Tunes Neuroserpin Physiological and Pathological Properties. Int. J. Mol. Sci. 2020, 21, 3235 10.3390/ijms21093235. PMC7247563 32375228

[ref52] Cale J. M.; Li S.-H.; Warnock M.; Su E. J.; North P. R.; Sanders K. L.; Puscau M. M.; Emal C. D.; Lawrence D. A. Characterization of a novel class of polyphenolic inhibitors of plasminogen activator inhibitor-1. J. Biol. Chem. 2010, 285, 7892–7902. 10.1074/jbc.m109.067967. 20061381 PMC2832939

[ref53] Huang R.-y.; Pei L.; Liu Q.; Chen S.; Dou H.; Shu G.; Yuan Z.-x.; Lin J.; Peng G.; Zhang W.; Fu H. Isobologram Analysis: A Comprehensive Review of Methodology and Current Research. Front. Pharmacol. 2019, 10, 1222 10.3389/fphar.2019.01222. 31736746 PMC6830115

