# Supplementary material for: Innovative Non-PrP-Targeted Drug Strategy Designed to Enhance Prion Clearance
Source: J Med Chem. 2022 Jun 30;65(13):8998–9010. doi: 10.1021/acs.jmedchem.2c00205 (PMC9289883; doi:10.1021/acs.jmedchem.2c00205)

Supporting Information

An innovative non-PrP-targeted drug strategy designed to enhance prion clearance

Arianna Colini Baldeschi [1, §], Marco Zattoni [1], Silvia Vanni [1, †], Lea Nikolic [1], Chiara Ferracin [1], Giuseppina La Sala [2, #], Maria Summa [3], Rosalia Bertorelli [3], Sine Mandrup Bertozzi [4], Gabriele Giachin [5], Paolo Carloni [6, 7, 8], Maria Laura Bolognesi [9], Marco De Vivo [2] and Giuseppe Legname [1, *]

AUTHOR ADDRESS

1 Laboratory of Prion Biology, Department of Neuroscience, Scuola Internazionale Superiore di Studi Avanzati (SISSA), Via Bonomea 265, 34136 Trieste, Italy

2 Molecular Modeling & Drug Discovery Lab, Istituto Italiano di Tecnologia, Via Morego 30, 16163 Genoa, Italy

3 Translational Pharmacology, Istituto Italiano di Tecnologia, Via Morego 30, 16163 Genoa, Italy

4 Analytical Chemistry Lab, Istituto Italiano di Tecnologia, Via Morego 30, 16163 Genoa, Italy

5 Department of Chemical Sciences (DiSC), University of Padua, Via F. Marzolo 1, 35131 Padova, Italy

6 Institute for Advanced Simulations (IAS)-5/Institute for Neuroscience and Medicine (INM)-9, , "Computational Medicine" Forschungszentrum Jülich, 52428 Jülich, Germany

7 Institute for Neuroscience and Medicine (INM)-11, "Molecular Neuroscience and Neuroimaging " Forschungszentrum Jülich, 52428 Jülich, Germany

8 Department of Physics, RWTH-Aachen University, Aachen, Germany

9 Department of Pharmacy and Biotechnology, University of Bologna, Via Belmeloro 6, 40126 Bologna, Italy

Corresponding Author

* Laboratory of Prion Biology, Department of Neuroscience, Scuola Internazionale Superiore di Studi Avanzati (SISSA), via Bonomea 265, 34136 Trieste, Italy. Email: legname@sissa.it

Present Addresses

§ Institute of Biomedicine, Department of Pathology and Experimental Therapeutics, Bellvitge University Hospital-IDIBELL, Barcelona, Spain.

† Osteoncology and Rare Tumors Center, IRCCS Istituto Romagnolo per lo Studio dei Tumori (IRST) "Dino Amadori", Meldola, Italy.

# Medicinal Chemistry, Research and Early Development, Cardiovascular, Renal and Metabolism (CVRM), BioPharmaceuticals R&D, AstraZeneca, Gothenburg, Sweden.

Table of contents

Table S1........ S3

Figure S1........ S7

Figure S2........ S8

Figure S3........ S9

Figure S4........ S10

Figure S5........ S11

Figure S6........ S12

Figure S7........ S13

Figure S8........ S14

Figure S9........ S15

Figure S10........ S16

Figure S11........ S17

Figure S12........ S18

Purity of compound 5........ S19

CODE	COMMERCIAL NAME	IUPAC NAME	CAS
A	ARN8755_Z_01	3-[[(5,5-dioxido-6H-dibenzo[c,e][1,2]thiazin-9-yl)amino]methyl]-7-methyl-2(1H)-Quinolinone	1026706-74-0
B	ARN5608_Z_01	2-(3-Oxo-2,3-dihydro-1H-isoindol-1-yl)-N-[2-(5-p-tolyl-2H-[1,2,4]triazol-3-yl)-ethyl]-acetamide	1214449-38-3
C	ARN2053_Z_01	1-(2-Fluoro-phenyl)-3-[1-(5-pyridin-3-yl-2H-[1,2,4]triazol-3-yl)-ethyl]-urea	1000355-25-8
D	ARN5896_Z_01	3-{[2-(3,5-Dimethyl-pyrazol-1-yl)-ethylamino]-methyl}-5-phenyl-1H-pyridin-2-one	1381725-39-8
E	ARN5158_Z_01	2-(6-chloro-2-oxo-1,3-benzoxazol-3-yl)-N-[(3-phenyl-1,2,4-oxadiazol-5-yl)methyl]acetamide	1214533-39-7
F	ARN2996_J_01	(3,5-Dimethyl-1H-indol-2-ylmethyl)-methyl-(1-methyl-2-pyridin-2-yl-ethyl)-amine	1287460-72-3 (cas of neutral form)
G	ARN2356_Z_01	[5-Amino-1-(4-fluoro-phenyl)-1H-pyrazol-4-yl]-(1H-pyrrol-2-yl)-methanone	1381295-34-6

H	ARN2357_Z_01	[5-Amino-1-(2,4-difluoro-phenyl)-1H-pyrazol-4-yl]-(1H-pyrrol-2-yl)-methanone	1381407-09-5
I	ARN10184_A_01	5-[2-(3-Chloro-phenyl)-ethyl]-3-{[(piperidin-4-ylmethyl)-amino]-methyl}-pyrazolo[1,5-a]pyrimidin-7-ol	2058940-45-5 (cas of neutral form)
L	ARN1510_Z_01	4'-Fluoro-biphenyl-3-carboxylic acid (1H-imidazol-2-ylmethyl)-amide	1381337-70-7
M	ARN5864_Z_01	1-(5-Pyridin-3-yl-2H-[1,2,4]triazol-3-ylmethyl) -3-(2-trifluoromethyl-phenyl)-urea	1000354-89-1
N	ARN8628_Z_01	5-Isopropyl-2H-pyrazole-3-carboxylic acid [2-(5-pyridin-3-yl-2H-[1,2,4]triazol-3-yl)-ethyl]-amide	1029834-71-6
O	ARN5863_Z_01	1-(3-Methoxy-phenyl)-3-(5-pyridin-3-yl-2H -[1,2,4]triazol-3-ylmethyl)-urea	1000353-70-7
P	ARN11880_Z_01	3-(5-Chloro-1-methyl-1H-benzoimidazol-2-yl) -N-(3-[1,2,4]triazol-1-ylmethyl-phenyl)-propionamide	1214586-09-0
Q	ARN1609_Z_01	(5-Fluoro-1H-indol-2-yl)-(3-imidazo[4,5-b]pyridin-3-yl-pyrrolidin-1-yl)-methanone	1381040-70-5 (racemate)
R	ARN9565_Z_01	1-[1-(4-Fluoro-phenyl)-1H-pyrazol-4-ylmethyl] -4-thiazol-2-yl-piperazine	1214413-13-4

S	ARN10145_Z_01	Benzyl-(7-methoxy-2H-chromen-3-ylmethyl)<br>-(6-methyl-pyridin-2-ylmethyl)-amine	1214435-09-2
T	ARN11752_Z_01	1-Pyrimidin-2-yl-piperidine-4-carboxylic acid (1-methyl-1H-imidazol-2-ylmethyl)-amide	2059666-43-0
U	ARN8304_Z_01	2-(3,5-Dimethyl-pyrazol-1-yl)-N-<br>[2-(5-pyridin-3-yl-2H-[1,2,4]triazol-3-yl)-<br>ethyl]-acetamide	958609-69-3
1	ARN11457_Z_01	[4-(5-Amino-1-p-tolyl-1H-pyrazole-4-<br>carbonyl)-piperidin-1-yl]-<br>(4-methyl-thiazol-5-yl)-methanone	1381325-24-1
2	ARN11512_Z_01	(5-Amino-1-p-tolyl-1H-pyrazol-4-yl)-<br>(1-cyclobutanecarbonyl-pyrrolidin-3-yl)-<br>methanone	1381388-32-4
3	ARN12643_Z_01	(5-Amino-1-phenyl-1H-pyrazol-4-yl)<br>-(1H-indol-3-yl)-methanone	1015939-70-4
4	ARN12924_Z_01	[4-(5-Amino-1-phenyl-1H-pyrazole-4-<br>carbonyl)<br>-piperidin-1-yl]-isoxazol-5-yl-methanone	1381478-56-3
**5**	ARN1468_Z_01	[5-Amino-1-(4-trifluoromethoxy-phenyl)-<br>1H-pyrazol-4-yl]-piperidin-4-yl-methanone	1381459-14-8
6	ARN1585_Z_01	1-{4-[5-Amino-1-(3-fluoro-phenyl)-<br>1H-pyrazole-4-carbonyl]-piperidin-1-yl}-<br>ethanone	1381429-03-3

7	ARN2275_Z_01	1-(4-Fluoro-phenyl)-1H -pyrazole-4-carboxylic acid (tetrahydro-pyran-3-yl)-amide	958625-69-9
8	ARN3623_Z_01	N-[(3,5-dimethyl-1H-pyrazol-4-yl)methyl]-1-(4-fluorophenyl)-1H-Pyrazole-4-carboxamide	1287426-60-1

Table S1. Compounds. The table lists code, commercial name, IUPAC name, and CAS number of each compound used in the present study

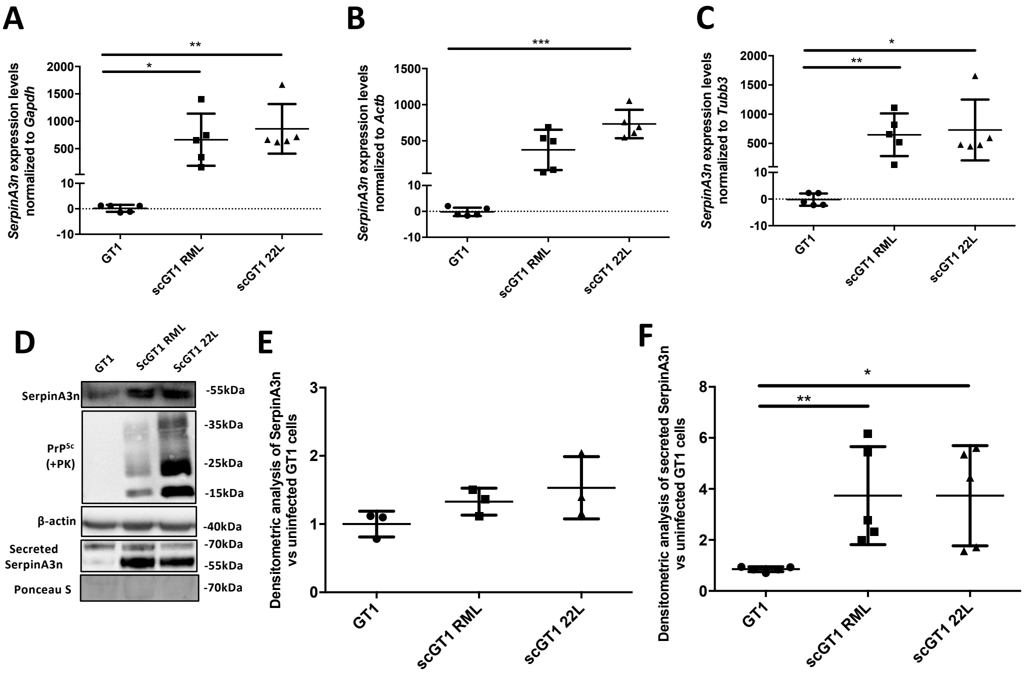

Figure S1. SerpinA3n expression in uninfected and prion-infected GT1 cell lines. A, **B**, **C** Gene expression analysis of SerpinA3n in RML- (n=5) and 22L-infected GT1 cell lines (n=5) compared to uninfected cells (n=5), normalized on *Gapdh* (A), *Actb* (B) and *Tubb3* (C) as reference genes. The relative expression ratio (fold change, FC) was calculated using the $2^{-\Delta\Delta C_T}$ method. ΔC_T was calculated by subtracting the C_T of the reference genes (*Gapdh*, *Actb*, *Tubb3*) from the C_T of the target one (*SerpinA3n*). $\Delta\Delta C_T$ values were obtained with the ΔC_T of each sample from the infected cell group (either with RML or 22L), minus the mean ΔC_T of the population of calibrator samples (uninfected cells). **D** Representative WB image of intracellular and secreted SerpinA3n on uninfected and RML- and 22L-infected GT1. Ponceau staining of the membrane for secreted SerpinA3n was used as a protein loading control. SerpinA3n and β-actin were developed on the same membrane, sequentially. PrP^{Sc} signal was developed on another membrane after PK-digestion of cell lysates. WB image has been cropped to improve the clarity of the signal. Molecular weight was represented on the right (kDa). **E** Densitometric analysis of intracellular SerpinA3n levels normalized on β-actin WB signal between uninfected (n=3) and RML- (n=3) and 22L-infected GT1 cell lines (n=3). **F** Densitometric analysis of secreted SerpinA3n levels WB signal between uninfected (n=5) and RML- (n=5) and 22L-infected GT1 cell lines (n=5). Statistical significance was performed using the Kruskal-Wallis test with Dunn's multiple comparisons compared to uninfected cells. *p<0.05, ** p<0.01.

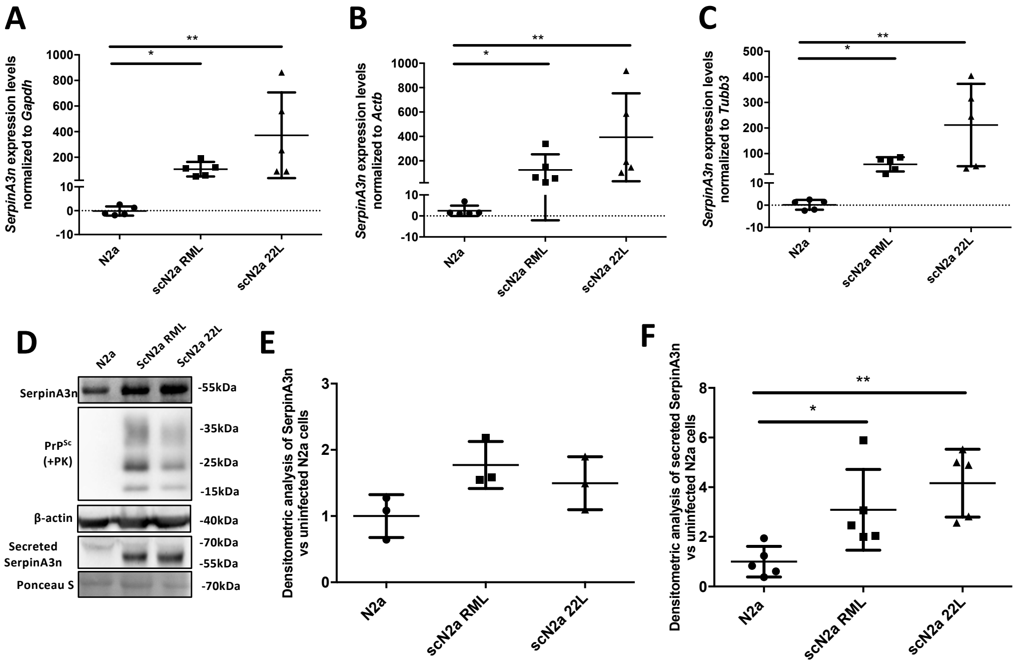

Figure S2. SerpinA3n expression in uninfected and prion-infected N2a cell lines. A, **B**, **C**. Gene expression analysis of SerpinA3n in RML- (n=5) and 22L-infected N2a cell lines (n=5) compared to uninfected cells (n=5), normalized on *Gapdh* (A), *Actb* (B) and *Tubb3* (C) as reference genes. The relative expression ratio (fold change, FC) was calculated using the $2^{-\Delta\Delta C_T}$ method. ΔC_T was calculated by subtracting the C_T of the reference genes (*Gapdh*, *Actb*, *Tubb3*) from the C_T of the target one (*SerpinA3n*). $\Delta\Delta C_T$ values were obtained with the ΔC_T of each sample from the infected cell group (either with RML or 22L), minus the mean ΔC_T of the population of calibrator samples (uninfected cells). **D** Representative WB image of intracellular and secreted SerpinA3n on uninfected and RML- and 22L-infected N2a. Ponceau staining of the membrane for secreted SerpinA3n was used as a protein loading control. SerpinA3n and β-actin were developed on the same membrane, sequentially. PrP^{Sc} signal was developed on another membrane after PK-digestion of cell lysates. WB image has been cropped to improvthe e clarity of the signal. Molecular weight was represented on the right (kDa). **E** Densitometric analysis of intracellular SerpinA3n levels normalized on β-actin WB signal between uninfected (n=3) and RML- (n=3) and 22L-infected N2a cell lines (n=3). **F** Densitometric analysis of secreted SerpinA3n levels WB signal between uninfected (n=5) and RML- (n=5) and 22L-infected N2a cell lines (n=5). Statistical significance was performed using the Kruskal-Wallis test with Dunn's multiple comparisons compared to uninfected cells. *$p<0.05$, ** $p<0.01$.

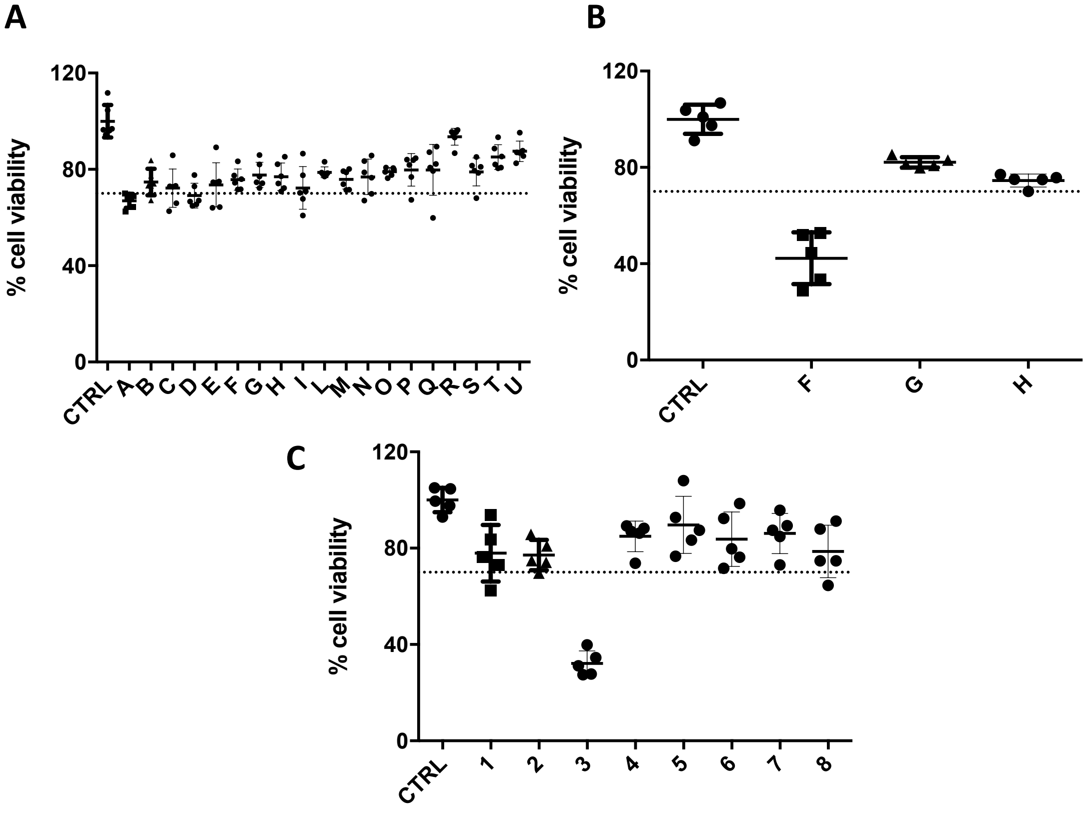

Figure S3. Cell viability assay of tested molecules. A MTT analysis of ScGT1 RML treated with the vehicle (CTRL) or the drugs (compounds A-U) at 20µM. **B** MTT analysis of ScGT1 RML treated with the vehicle (CTRL) or the drug (F, G, and H) at 40µM. **C** MTT analysis of ScGT1 RML treated with the vehicle (CTRL) or the drug (compounds 1-8) at 20µM. The experiment has been performed in six technical replicates.

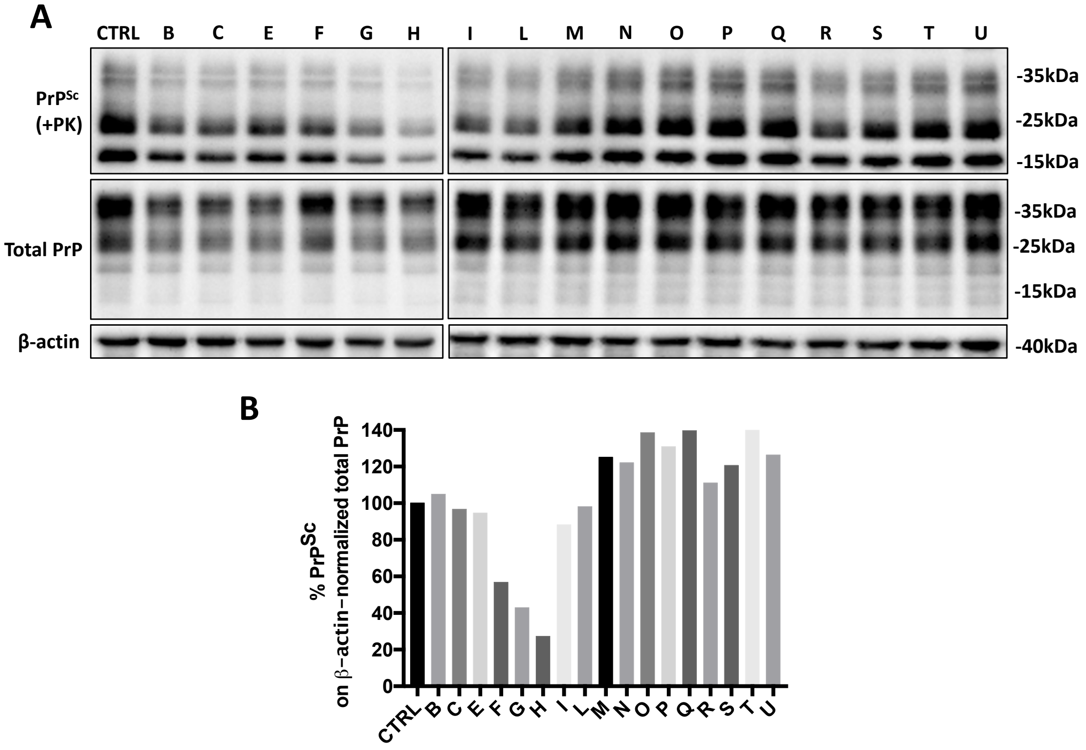

Figure S4. Anti-prion effect of first library molecules. A Western blotting of PrP^{Sc}, Total PrP and β-actin in lysates from ScGT1 RML treated with vehicle (CTRL) or drug (compounds A-U) at 20μM. Molecular weight is represented on the right (kDa). **B** densitometric analysis of normalized PrP^{Sc} levels in ScGT1 RML treated with the vehicle or the drug.

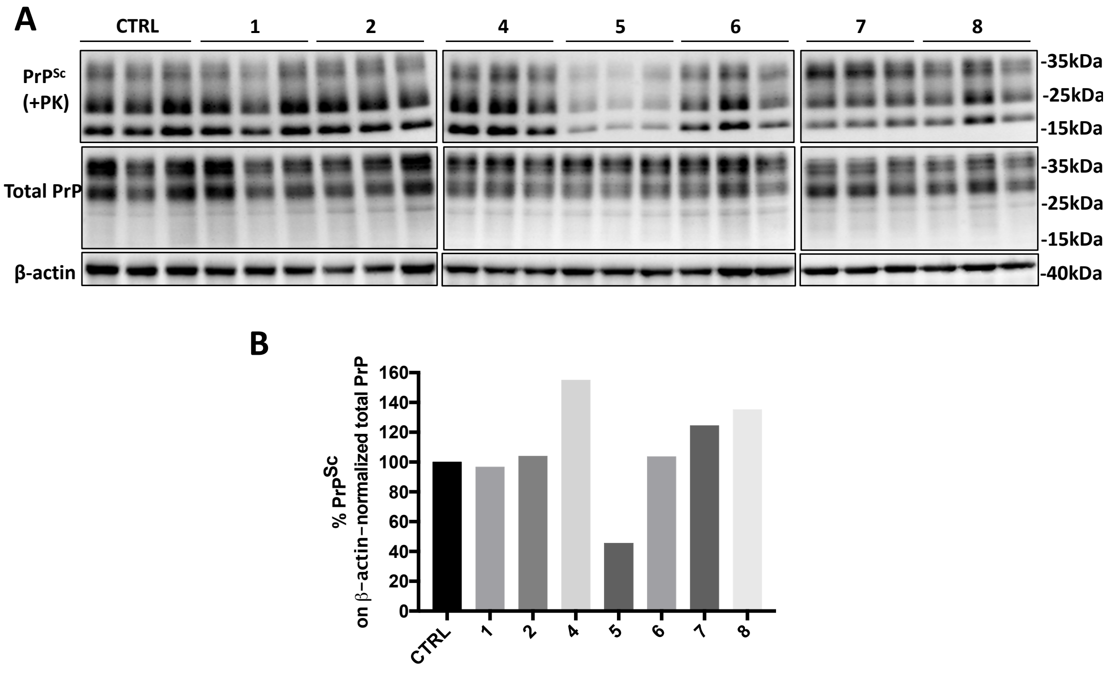

Figure S5. Anti-prion effect of second library molecules. A Western blotting of PrP^{Sc}, Total PrP and β-actin in lysates from ScGT1 RML treated with vehicle (CTRL) or drug (compounds 1-8) at 20μM. The experiment has been performed in triples. Molecular weight is represented on the right (kDa). **B** Densitometric analysis of normalized PrP^{Sc} levels in ScGT1 RML treated with the vehicle or the drug.

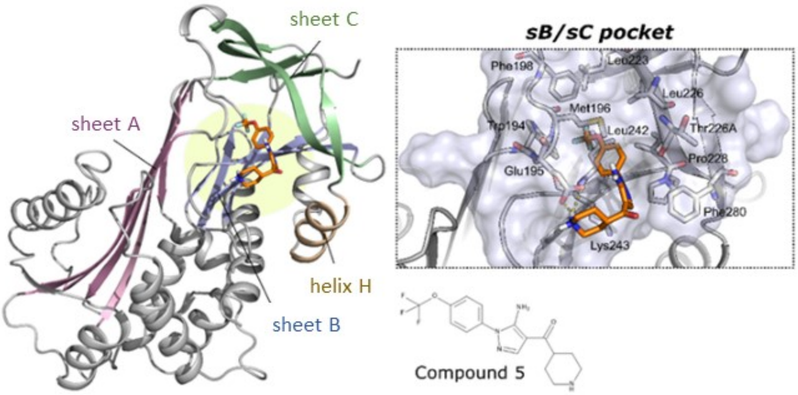

Figure S6. Binding mode of compound 5. Left: the structure of SerpinA3n (pdb: 1AS4) is shown in cartoon style, with beta-sheet A in pink, beta-sheet B in light blue, beta-sheet C in green, and the helix H in sand. Compound 5 is shown in orange sticks. The sB/sC pocket is highlighted with a lemon circle. Right: Close up on the sB/sC pocket. The latter is represented in grey surface. Compound 5 is represented as orange sticks. Residues in the binding pocket are shown in grey sticks.

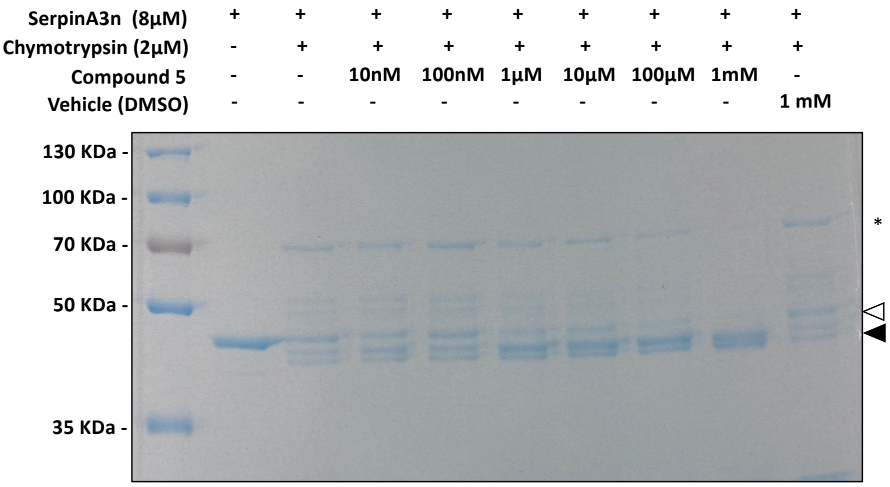

Figure S7. Compound 5 inhibitory activity on SerpinA3n-chymotrypsin complex formation. SDS-PAGE Coomassie blue staining of increasing concentrations (10nM to 1mM) compound 5 or vehicle (DMSO) incubated with SerpinA3n (8µM) and chymotrypsin (2µM), showing SDS-stable SerpinA3n-chymotrypsin complexes (asterisk), active SerpinA3n (white arrowhead), and cleaved SerpinA3n (black arrowhead).

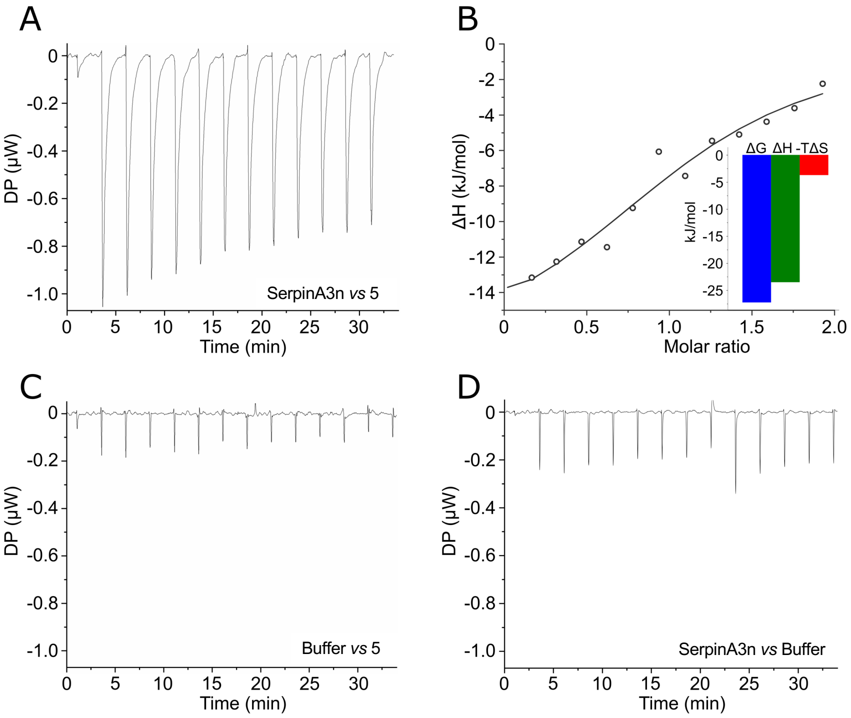

Figure S8. ITC measurements. A A representative raw titration data obtained from the titration of 16 μM SerpinA3n with 160 μM compound 5. **B** Wiseman plot of integrated data (black circles) and fitted isothermal binding curve. From two independent experiments, an apparent mean KD of 26 μM for the single macroscopic dissociation constant was obtained, with a 1:1 stoichiometry. In the inset, the signature shows a mean ΔG, ΔH and –TΔS of approximately -27.3, -23.5 and -3.73 kJ/mol, respectively. **C**, **D** Representative raw titration data obtained from the titration of 160 μM compound 5 (C), and 16 μM SerpinA3n (D) with buffer, as controls.

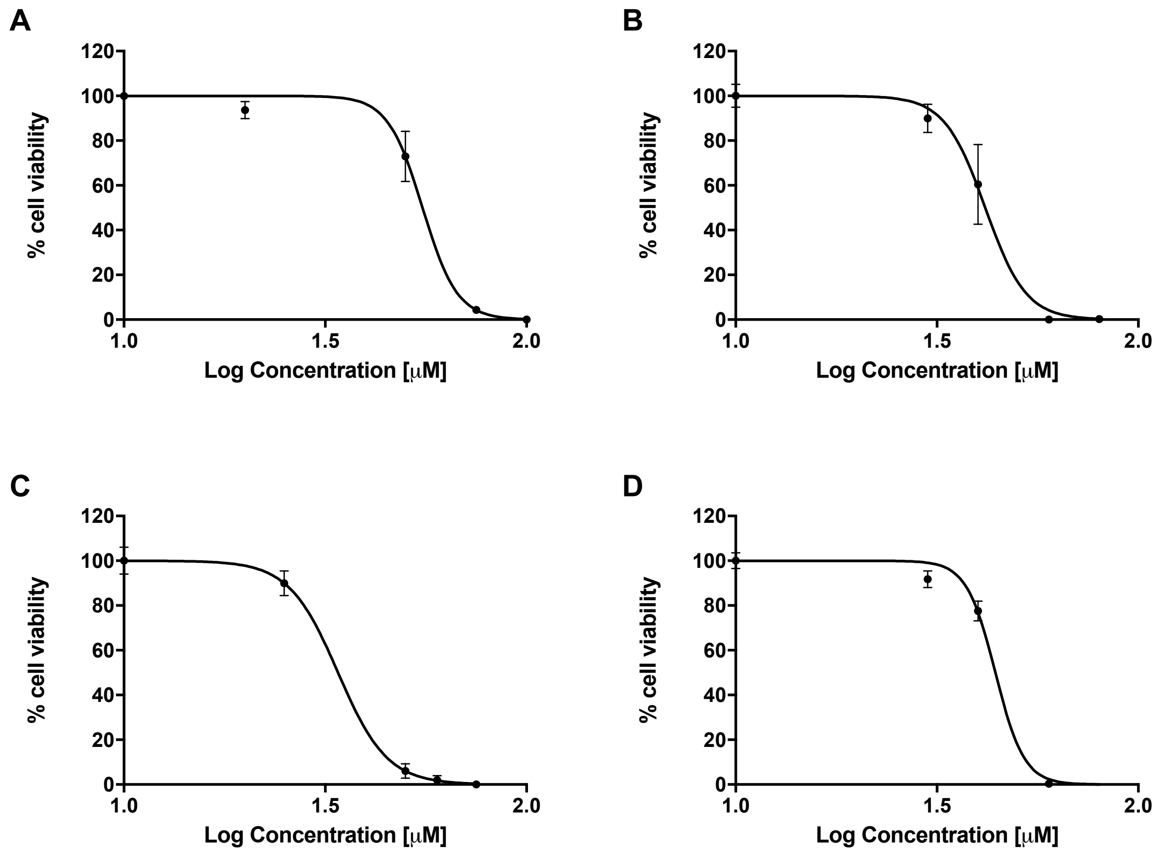

Figure S9. Dose-survival curve of compound 5 on RML- and 22L-infected GT1 and N2a cell lines. MTT analysis after 6 days treatment with increasing concentration of compound 5 in ScGT1 RML (A, LD50=55.17), ScGT1 22L (B, LD50=41.68) ScN2a RML (C, LD50=44.17) and ScN2a 22L (D, LD50=34.03) (n=3).

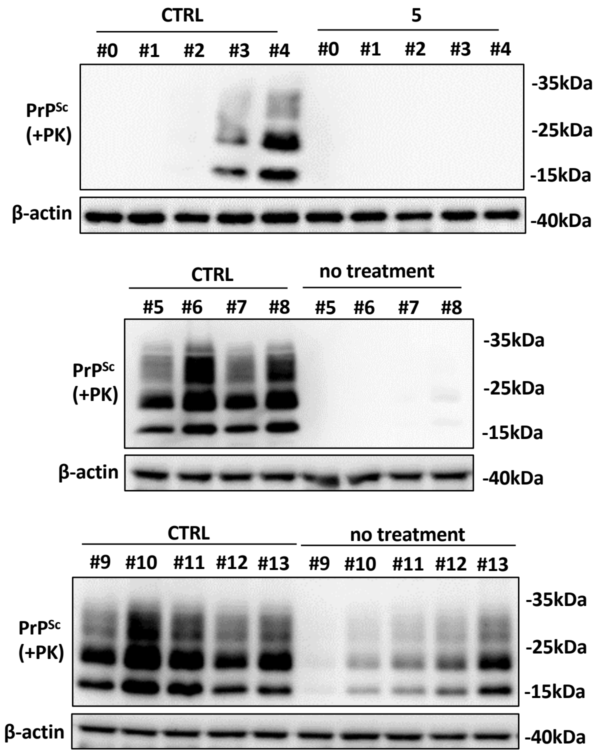

Figure S10. *De novo* infection with compound 5. Western blotting analysis of PrP^{Sc} in lysates from *de novo* infected GT1 cell line treated with vehicle (CTRL) or the drug (compound 5) for several passages at 20μM. β-actin has been performed as a loading control. Molecular weight is represented on the right (kDa).

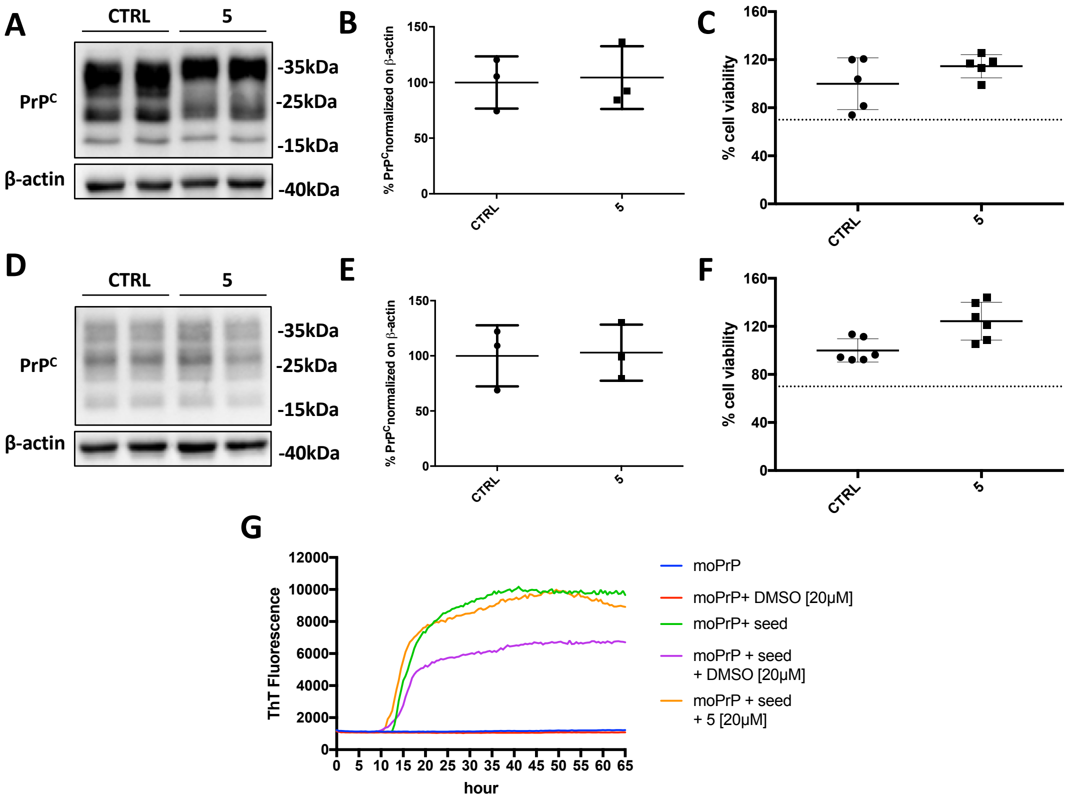

Figure S11. Cell viability assay and PrP^{C} amount in compound 5-treated not-infected cells and PrP^{C} aggregation propensity in presence of compound 5. A, D Representative Western Blot image of PrP^{C} and β-actin in lysates from GT1 (A) N2a (D) treated with vehicle (CTRL) or compound 5 at 20μM. Molecular weight is represented on the right (kDa). **B, E** Densitometric analysis of normalized PrP^{C} levels GT1 (B) and N2a cells (E) treated with the vehicle (CTRL) or compound 5. Experiments have been performed in duplicates (n=3). **C, F** MTT analysis of GT1 (C) and N2a (F) treated with the vehicle (CTRL) or compound 5 at 20μM. The experiment has been performed in six technical replicates. **G** RT-QuIC analysis of PrP-compound 5 interaction. The presence of "seed" (PrP^{Sc}) is responsible for an increased formation of ThT fluorescent PrP aggregates in presence (green line) or absence of compound 5 (orange line). Incubation of recombinant mouse PrP (moPrP) alone (blue line) or in presence of DMSO (red line) has been performed as negative control. A positive control is represented by moPrP in presence of DMSO to which "seed" has been added (purple line).

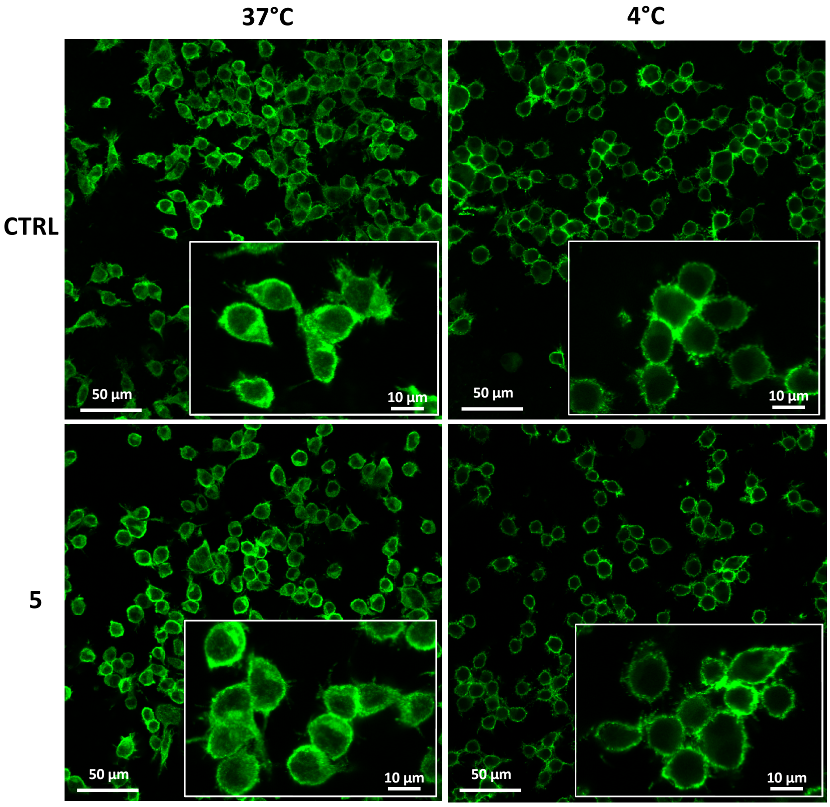

Figure S12. PrP^{C} localization upon compound 5 treatment. Immunofluorescence staining of PrP^{C} (experiment performed at 37°C) and membrane only PrP^{C} (experiment performed at 4°C, to block PrP recycling) in N2a cells treated with compound 5 compared to control cells (treated with vehicle only, DMSO).

Purity of compound 5

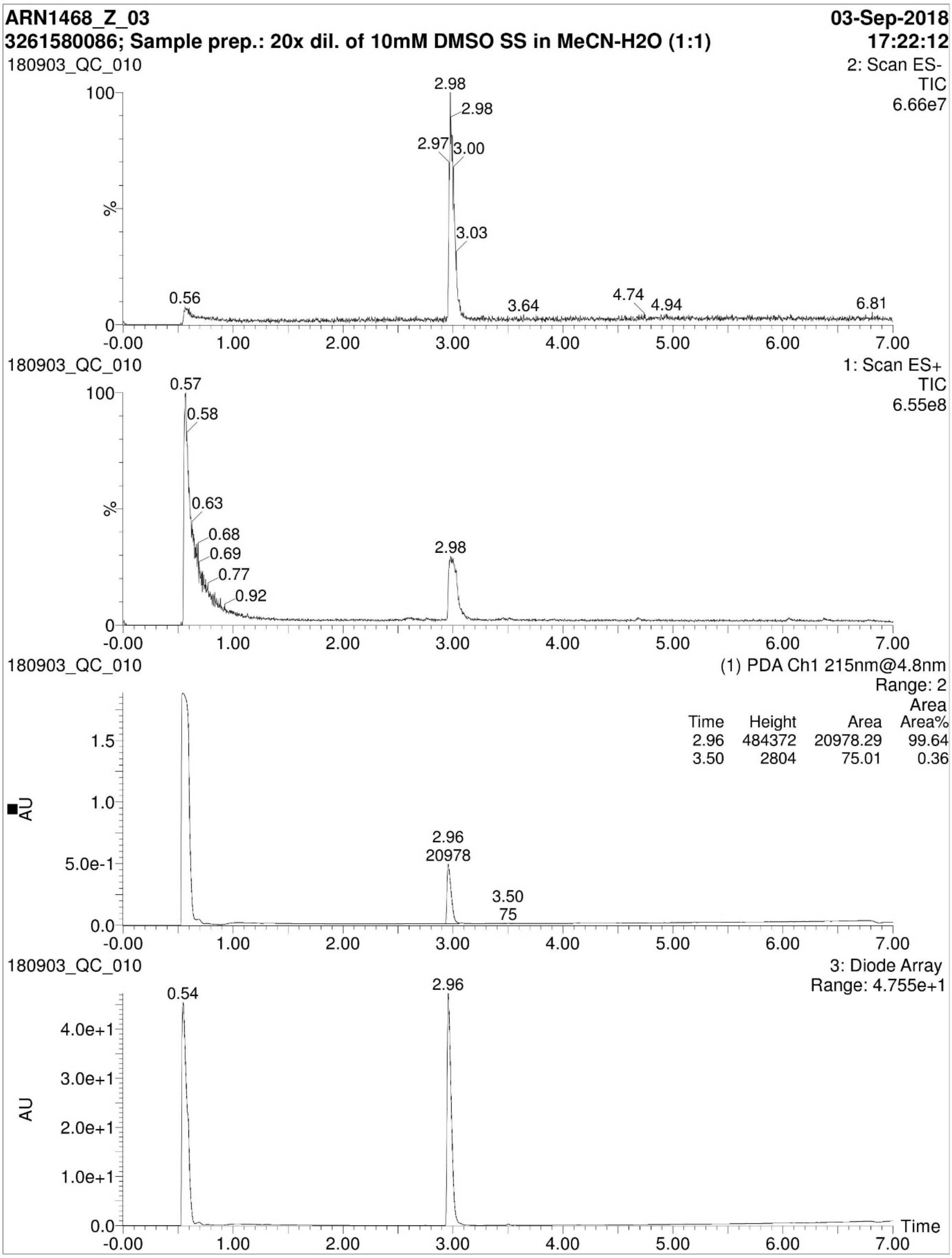

ARN1468_Z_03 **03-Sep-2018**

3261580086; Sample prep.: 20x dil. of 10mM DMSO SS in MeCN-H2O (1:1) **17:22:12**

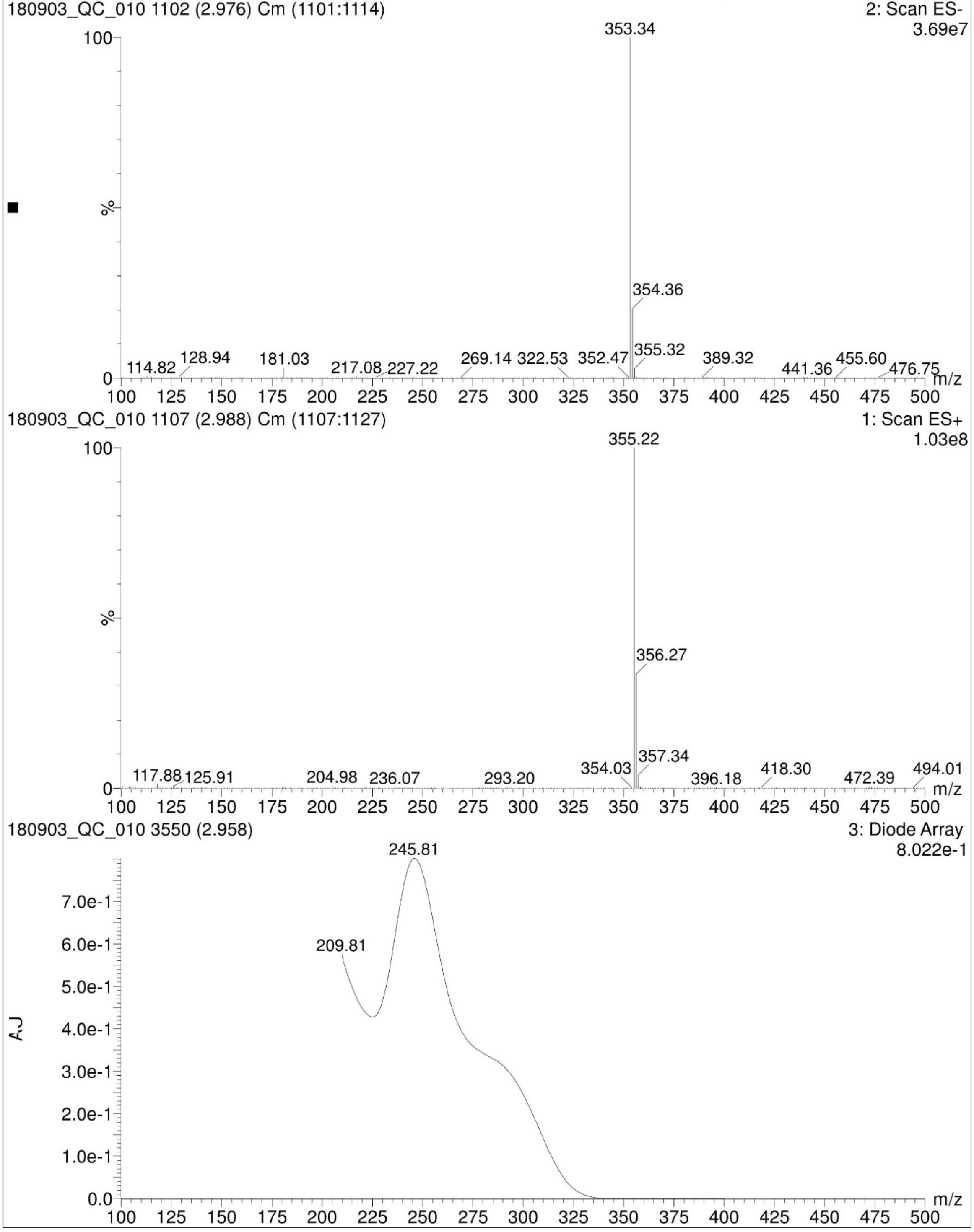

Supplement: Supplementary file 1 — jm2c00205_si_001.pdf [file jm2c00205_si_001.pdf]
